# Synchronous birth is a dominant pattern in receptor-ligand evolution

**DOI:** 10.1186/s12864-018-4977-2

**Published:** 2018-08-14

**Authors:** Anna Grandchamp, Philippe Monget

**Affiliations:** 0000 0001 2182 6141 grid.12366.30 PRC, UMR85, INRA, CNRS, IFCE, Université de Tours, F-37380 Nouzilly, France

**Keywords:** Ligand, Receptor, Phylogeny, Co-appearance

## Abstract

**Background:**

Interactions between proteins are key components in the chemical and physical processes of living organisms. Among these interactions, membrane receptors and their ligands are particularly important because they are at the interface between extracellular and intracellular environments. Many studies have investigated how binding partners have co-evolved in genomes during the evolution. However, little is known about the establishment of the interaction on a phylogenetic scale.

In this study, we systematically studied the time of birth of genes encoding human membrane receptors and their ligands in the animal tree of life. We examined a total of 553 pairs of ligands/receptors, representing non-redundant interactions.

**Results:**

We found that 41% of the receptors and their respective first ligands appeared in the same branch, representing 2.5-fold more than expected by chance, thus suggesting an evolutionary dynamic of interdependence and conservation between these partners. In contrast, 21% of the receptors appeared after their ligand, i.e. three-fold less often than expected by chance. Most surprisingly, 38% of the receptors appeared before their first ligand, as much as expected by chance.

**Conclusions:**

According to these results, we propose that a selective pressure is exerted on ligands and receptors once they appear, that would remove molecules whose partner does not appear quickly.

**Electronic supplementary material:**

The online version of this article (10.1186/s12864-018-4977-2) contains supplementary material, which is available to authorized users.

## Background

The co-evolution of genes encoding interacting molecules is a subject of intense study [1–4] because of the intriguing question of the modes of mutation and selection that act on two molecules simultaneously. In particular, the co-evolution of the binding motif has been well investigated [5]. These studies of co-evolution focused for example on the fitness [6, 7], on the conservation of the interaction [8–10], or on the evolution of the residues at the interface of the molecules [11–13]. While these studies on the coevolution of binding partners often require the integration of different disciplines (chemistry, evolution, biology), the establishment of the interaction from a phylogenetic point of view is less studied. Little is known for example about the origin and evolution of the different partners prior to their first interaction. Do the receptor and the ligand co-exist independently before they start to interact? Does the emergence of one partner favor the emergence of the second partner? If so, which tends to come first, the receptor or the ligand? The creation of new genetic material often relies on segmental duplication, or sometimes but more rarely on entire genome duplication [14–17]. Once a gene is born, either de novo for the first member of a family or by duplication of existing genes, the gene will be subjected to negative selection if it is not beneficial, and could even be lost by pseudogenisation [14]. If the gene belongs to a gene family, for example the glycoproteins FSH, LH and TSH and their receptors, the appearance of the first member of the family can be the result of an ancestral duplication of a gene that belongs to the superfamily (GPCR superfamily in this case), followed by several mutations leading to the current genes. The diversifications of GPCR families arose by multiple duplications [18, 19] However, it is only the acquisition of a novel function that will allow the maintenance of the newly duplicated gene.

In the case of interacting molecules, the appearance of genes coding for molecules included in a complex is more intricate [20]. For two molecules that will eventually interact, the appearance of one may be dependent on the appearance and conservation of the other. This may be the case, for example, when the presence of the first molecule is not advantageous as long as its partner has not yet appeared.

Asking the question: “In the absence of a ligand, what is the biological role of a receptor?”, Thornton [21] has shown that the first steroid receptor of the family, present in lamprey and supposed to be present in the common ancestor of vertebrates, was an oestrogen (that is, a steroid) receptor, and that several duplications led to other steroid receptors, specialized in other functions with other ligands. However, recent investigations suggest that the ancestral ligand for the ancestral steroid receptor was a molecule with a structure distinct from modern estrogen, an aromatized steroid with a side-chain, called paraestrol [22]. Yet the existence of receptors without partners, called orphan receptors, has also been frequently described [23], even though it is sometimes difficult to assess whether a receptor is a true orphan or its ligand is just unknown [24]. Interestingly, studies have demonstrated that orphan nuclear receptors were phylogenetically related, and older than the receptors with a known ligand [25, 26]. These authors have suggested that the receptor acquired its binding pocket during evolution. In contrast, more recently, the existence of an ancient common ligand of the nuclear receptor family was demonstrated [27], thus challenging the view that nuclear receptors could have evolved for extended periods of time without ligand.

The relative appearance of genes encoding protein partners is thus an open question. Furthermore, several types of interactions can be observed in living organisms, with different numbers of interacting partners [28–31], varying affinities [32], or different duration for the interaction [33], making the problem more complex.

Understanding the process that leads to functional interactions would help to understand how genes evolve to give rise to a binding pocket in receptors during evolution. With thousands of entirely sequenced genomes available in public databases, assessing when a functional gene appears in the tree of life is becoming a realistic challenge.

In our study, we collected a list of genes encoding human cell membrane receptors with their known ligands, and studied the timing of their respective appearance during evolution.

## Methods

### Implementation of the database

Our study is focused on human membrane receptors and human endogenous ligands, for which information was collected from several sources (Additional file 1). The genes encoding receptors whose ligands were not endogenous, such as olfactory receptors or taste receptors, were not considered. In 101 cases, the ligands resulted from a chain of synthesis that requires several enzymes (such as dopamine, serotonin, acetylcholine, etc.). In these cases, we considered the set of genes encoding the enzymes involved in the ligand synthesis. The number of genes encoding such enzymes varied between 1 and 4 genes.. Nuclear receptors and their ligands were not considered, owing to the large number of genes involved in the synthesis of the ligand (more than 15 genes can be involved). Ultimately, we built a list of 1479 pairs of genes encoding respectively a ligand and its membrane receptor, which is three times greater than can be found in the DIP (database of interacting proteins) database. We only used interactions confirmed by experimental assays. However we also repeated our calculations using a larger list of predicted interactions previously described by Ramilowski et al. [34] to make sure that the results would not be modified (Additional file 2). Ramilowski’s is the most comprehensive list in existence today. Better-known lists recording the complete interactome, such as StringDB [35], were not used, because they do not specify the nature of the interactions (ligand -receptor, substrate-enzyme) in the case of ligands receptors. Moreover, the ligands receptors interactions implemented in StringDb come from DIP database that was used in our list.

### Phylogenetic study

In order to determine the time of appearance of each gene, we focused our study on the animal tree of life [36], and on the phylogenetic trees of animal sequences available in Ensembl [36]. We selected 10 phylogenetic branches as possible intervals where a gene may have appeared. The branch of appearance of a gene refers to the branch that include all the taxonomic groups in which the gene is present and functional today. For example, if a gene was present in several taxonomic groups such as mammals, reptiles, amphibians, and not in other groups, we consider that the functional ancestor of the gene appeared in Tetrapoda. The absence of a gene in taxonomic groups which diverged before Tetrapoda could be due to a loss of the genes in the species of this group that are available in Ensembl. For taxonomic groups in which there were few species in Ensembl (see Additional file 2), the gene was looked up in Refseq (Genbank) using tBLASTn [37] to make sure it could not be found in other species.

**Fig. 1:**
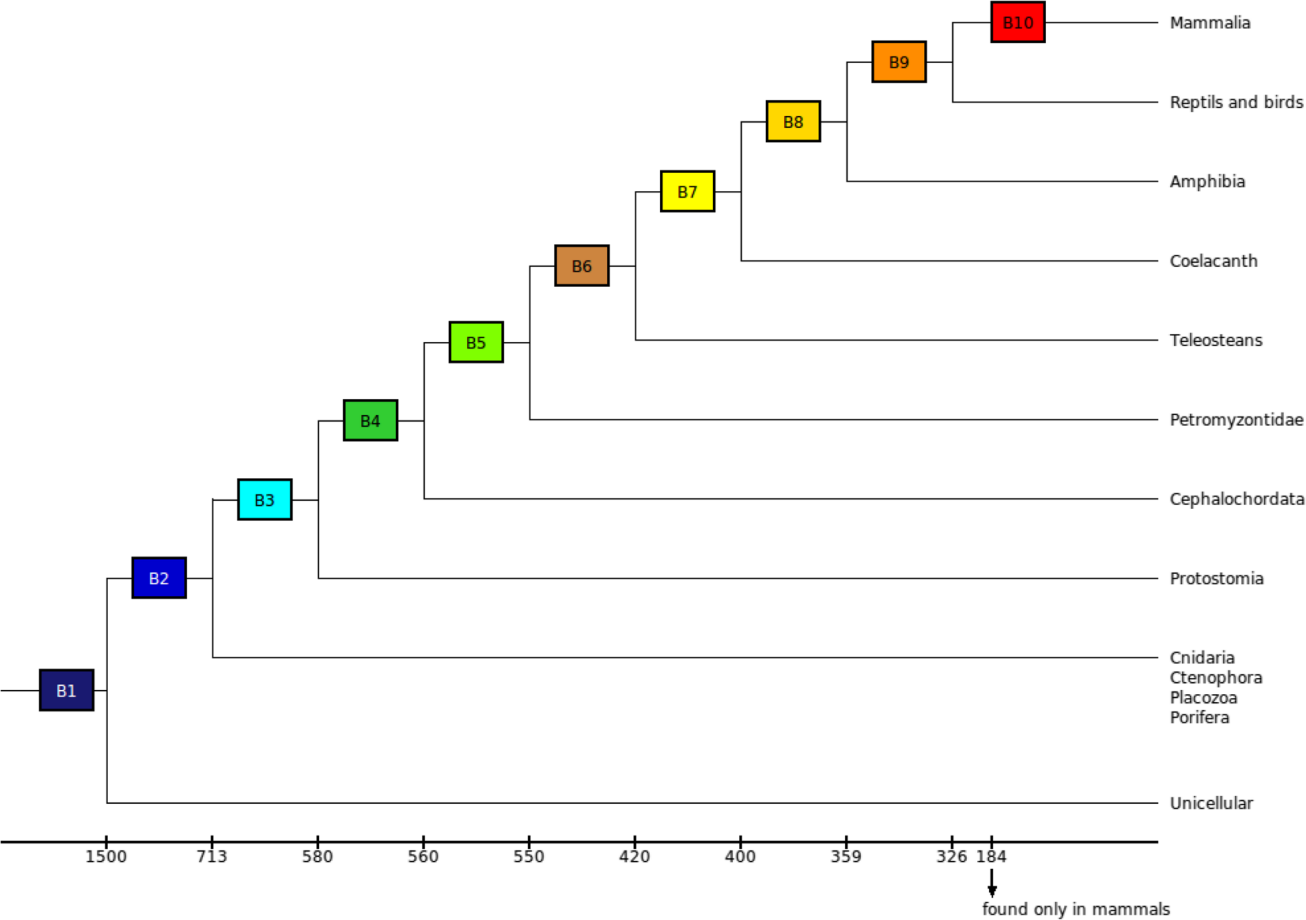
Definition of branches in the animal tree of life. Rectangles represent the 10 defined branches of appearance for the proteins (B1 to B10). The estimated time of emergence of the branches is indicated under the tree

We defined 10 phylogenetic branches (Fig. 1): branch 1 is ancestral to yeast and multicellular organisms, whose emergence is dated about 1500 million years (my), branch 2 is ancestral to Metazoa (~ 713 my), therefore excluding unicellular organisms, branch 3 is ancestral to bilaterians (~ 580 my), branch 4 is ancestral to Chordates (~ 560 my), branch 5 is ancestral to vertebrates (~ 550 my), branch 6 is ancestral to Teleosts (~ 420 my), branch 7 is ancestral to Sarcopterygians (~ 400 my), branch 8 is ancestral to Tetrapods (~ 359 my), branch 9 is ancestral to Amniotes (~ 326 my), and branch 10 is ancestral to mammals (~ 184 my). At the base of the metazoan tree, we decided to define only one branch (branch 2) that would be ancestral to the Placozoa, the Porifera, the Ctenophora and the Cnidaria, because their phylogeny is still being discussed [36]. Indeed, we estimated that the merging of these groups may introduce a smaller bias in our study than considering each separately.

The ten branches defined are separated by distinct time steps. Indeed, some branches have diverged within short time steps, as for example the vertebrate branch, which diverged from the non vertebrate chordates 550 my ago, and the branch of the chordates, which diverged from the unchurched 560 my ago. So there is a short time step of 10 my between these two branches. On the other hand, there is a time step of 110 my between the branch of the vertebrates and the branch of teleosts, which diverged from non-teleost vertebrates 420 my ago. These different time step were taken into account in our statistical model (see after).

The choice to rely on such wide time gaps has allowed us to highlight the possibility for one of the interacting partners to remain maintained during the evolution over a broad time without the presence of its current partner. However, this choice made it impossible to precisely date the moment of appearance of the gene in the branch.

We then determined in which branch the genes encoding each receptor and ligand appeared. The phylogenetic trees were recovered from the ENSEMBL database v82 [38]. We complemented the branch of the first Metazoans (branch 2) using the Ensembl metazoan database (http://metazoa.ensembl.org/index.html), thus adding 71 genomes. For the trees that rooted in non chordate species, we identified and selected the corresponding genes in the Ensembl Metazoa database. For each gene in our list, its branch of appearance was annotated. A total of 145 species were considered in the phylogenetic trees (Additional file 1).

It is now known that two rounds of complete duplication are at the origin of the vertebrate genomes [39]. In the case of ligands and receptors, it is expected that some ligands and receptors that appeared in non-chordates are therefore present in four copies in vertebrates. However, this is not the case for most of the gene families, less than 5% of duplicate gene families remaining in duplicate [40]. So gene families rarely present 4 duplicate copies of the ancestral gene. Nevertheless, we took into account this complete duplication in our study. For each copy resulting from the duplication, the root we considered was the one given by the Ensembl algorithms. In most cases, for a receptor having duplicated in several copies, the root given by Ensembl is the branch of appearance of the first receptor. It is the same for the ligands, whose root will mainly be the ancestral root.

However, there are less frequent cases of some genes with strong divergence on one of the duplicates just after duplication. This is the case if an ancestral receptor is duplicated, and one of the duplicates diverges very specifically to bind a new ligand.This is for example the case for ephrin receptors. Some of these receptors are present in non-chorded animals, along with their ligands, and some other of these receptors appeared in the vertebrate branch after the two duplications. The latter bind to the same ligands as the ancestral receptors. Thus, the first receptors of this family appeared at the same time as their ligands, when the other receptors of the family, resulting from complete duplication, appeared after their first ligand. We find an inverse case with integrins. Most of their receptors appeared in the first metazoans, as well as their ligands. That is not however the case with ITGAD, an integrin whose ligand appeared in vertebrates. Phylogeny does find an ortholog of ITGAD in non-chorded animals. In this rather special case, for most members of the integrin family, the first ligand appeared in the same branch, except for this particular gene whose first ligand appeared later.

During the course of our study, we realized that the majority of the members of a given family appeared in the same branch (to take the same example as above, FSH, LH and TSH, which belong to the same family, appeared at the same branch). However, it is not the case for all the families. For example, some genes evolve faster than others, such as the genes involved in immunity [4]. In such a case, the trees tend to give the same root for all the genes coding for interleukins because Ensembl trees are based on a very stringent alignment, whereas some of the subfamilies did not appear at the first root. All these trees were treated manually, to make sure that all the complicated situations would be taken into account. To reduce the number of possible incorrect datings, according to our defined branches, we took the sequences of all the species of Ensembl that branch to the oldest root of the tree, to verify by tblastn analysis if an older ancestor was present in the syntenic region. For example, if the tree included mammals, reptiles and amphibians, we took the sequences of the species corresponding to these taxonomic groups present in Ensembl. Then, t-blastn were performed (in Refseq of NCBI, [37]) on the genome of all the outgroup species descending from the node directly preceding (i.e. more ancient than) the root, according to our defined branches. Moreover, some genes are not annotated by their name. This fact could bias the Ensembl research. In fact, an ortholog of a gene of interest could be present in species that branch in a branch older than the root given by Ensembl, but not encountered in Ensembl because it is not annotated. We systematically used Mapviewer (https://www.ncbi.nlm.nih.gov/genome/gdv/) to examine the conservation of synteny in order to correct the phylogeny as previously described [41, 42]. BLAST and synteny conservation allowed us to correct 47 trees for which the gene was found to appear 1 branch earlier, and 16 trees for which the gene was found to appear 2 or 3 branches earlier. All of the 63 genes concerned were involved in immunity.

### Study of the birth of genes encoding the ligands and their receptors

**Fig. 2:**
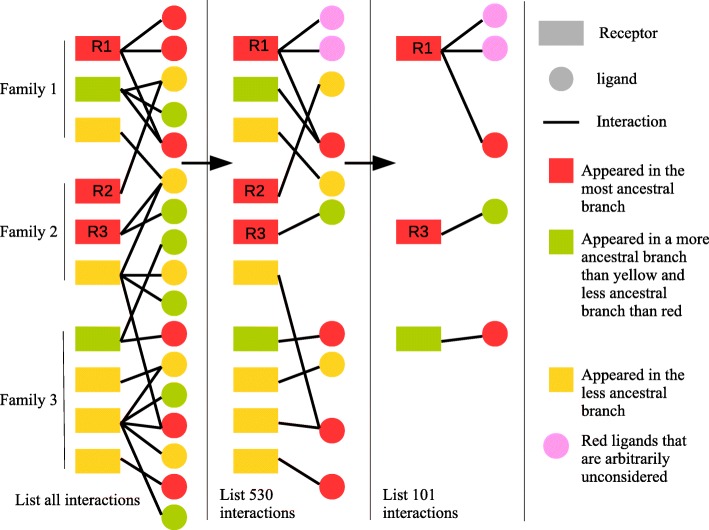
Schematic representation of the three list of interactions depicted in the article. The “complete list” represents the initial list of 1479 interactions. The receptors are grouped by family. Each receptor establishes interactions with one or more ligands. Here as an example, we have represented 22 interactions among the 1479 in the real list. In red are represented the molecules, ligands as receptors, appeared in the most ancestral branch. In green are represented the molecules that appeared in a less ancestral branch than the red molecules, and in yellow the molecules that appeared in a branch less ancestral than green molecules. The “Non redundant interactions” represents the reduction of the global list of 1479 interactions to 553 non-redundant interactions, by removing all the ligands that are not the most ancestral ones. In case several ligands appeared at the same time (R1 receptor), we consider only one ligand, by arbitrarily choosing one of them. The nature of the ligand does not matter because it is only the branch of appearance (common to all) that concerns us. In this diagram, the list of 22 redundant interactions is reduced to 10 interactions (one per receptor). The “One interaction by family” represents the reduction of the list of 553 non-redundant interactions into a list of 113 interactions. To make this reduction, we takes in every receptor family the first receptor that appeared, with its first ligand. In the case of family 2 of the diagram, we note that two receptors appeared at the same time and in the most ancestral branch. In this case, we choose the receptor whose first ligand appeared. Here, it is R3 that is chosen rather than R2. In this list we go from 10 interactions to 3, as many interactions as there are families

The main point of the experiment at this point was the reshuffling of our list of 1479. This reduction aimed to consider only the first ligand(s) that appeared for each receptor, and vice versa. Indeed, many receptors (more than 75%) have several ligands. These ligands often belong to the same family, but this is not always the case (i.e. LIFR, vldlr etc.). For each receptor, when it appeared in a phylogeny, we tried to determine whether it had a ligand to interact with as soon as it appeared (it is the case if at least one of its current ligands appeared in a preceding branch), if the appearance of interacting ligands took place in the same branch (it is the case if at least one of its current ligands appeared in the same branch and another one later), or if at the time of appearance of the receptor, none of the ligands was still present (i.e. the first ligand(s) appeared in later branches). The interactions with the other ligands, those that appeared later, were not considered here, since they concern coevolution. Our list of 1479 interactions was thus reduced to a list which included the 553 receptors of the first list, accompanied by the moment of appearance of their first ligand(s). Moreover, to ensure that these few families did not introduce any bias, we also set up a list, including only the first receptor which appeared in each family, with its first ligand. We obtained a list of only 113 pairs, with the earliest receptor of the 113 families and their first ligand. Thus, such a list, although much less precise and including less data, allowed us to ensure that any misidentification of the moments of appearance of the molecules resulting from duplications in the multigene families would be discarded (Fig. 2, Additional file 2).

Concerning the 553 interactions, 101 receptors bound only with ligands that were not peptides, but molecules generated by a chain of synthesis involving several enzymes. Among these 101 pairs, for 49 of the 101 pairs in which the ligands were the result of a chain of synthesis involving several enzymes, all the genes encoding the enzymes appeared in the same branch, which we considered to be the branch of appearance of the ligand. For the remaining 52, we only considered the branch of appearance of the most recent gene involved in the synthesis, considering that the resulting ligand could not be present without all the enzymes necessary for its synthesis.

Each pair of ligand/receptor was classified as follows: LB-Ligand Before, the gene coding for the first ligand appeared before the gene coding for the receptor; LS-Ligand Synchronous, the gene coding for the first ligand appeared in the same branch as the gene coding for the receptor; LA-Ligand After, the gene coding for the first ligand appeared after the gene coding for the receptor. The distribution of the pairs in each category was analyzed for the complete list (553 pairs), and with two other configurations grouping receptors by families, to make sure that the results are not impacted by possible duplication biases within families, and by removing the ligand whose synthesis involved several enzymes. The list we built only contains interactions verified by experiments. To examine if adding predicted interactions would affect our data, we also repeated the analysis using the predicted interactions that involved our receptor, using the list of Ramilowski [34], although the list was filtered to remove genes coding for G proteins and other proteins that are not ligands (see Additional file 1).

### Model of comparison

We conducted a test to estimate whether the distribution of the pairs in the three categories was different from what would be expected if both partners appeared independently.

To this end, the proportion of all human genes that appeared within each of our delimited branches was assessed by counting the number of roots of all 19,928 human gene trees in each branch. The time that has elapsed within the branches was taken into account by weighting the number of genes that appeared in each of them. We also did the tests without taking into account this weighting, which gave the same statistical result (Additional file 2). This frequency distribution enabled us to compute the null distribution of ligands appearing before (LB), after (LA), and at the same time (LS) as their receptor:

$$ {\mathrm{L}}_{\mathrm{B}}=\sum \limits_{\mathrm{b}1=2}^{\mathrm{b}1=10}\left(\sum \limits_{\mathrm{b}2=1}^{\mathrm{b}2=\mathrm{b}1-1}{\mathrm{R}}_{\mathrm{b}2}\times {\mathrm{F}}_{\mathrm{b}2}\right) $$

$$ {\mathrm{L}}_{\mathrm{s}}=\sum \limits_{\mathrm{b}=1}^{\mathrm{b}=10}{\mathrm{R}}_{\mathrm{b}}\times {\mathrm{F}}_{\mathrm{b}} $$

$$ {\mathrm{L}}_{\mathrm{A}}=\sum \limits_{\mathrm{b}1=1}^{\mathrm{b}1=9}\left(\sum \limits_{\mathrm{b}2=\mathrm{b}1+1}^{\mathrm{b}2=10}{\mathrm{R}}_{\mathrm{b}2}\times {\mathrm{F}}_{\mathrm{b}2}\right) $$

With Rb the number of receptors observed in branch b and Fb the frequency of protein appearance in branch b. The branches, that are b symbols, are the branches franked 1 to 10. In eqs. LA and LB, b1 corresponds to the variation in branches in the first sum, and b2 to that in the second one. b2 may vary independently of b1.

The difference between the observed and the theoretical distribution was assessed with a Pearson’s chi-squared test. The test was performed in the 4 configurations: with all receptors, with receptors grouped by family, with all receptors but removing the ligands that result from a chain of synthesis in which the enzymes involved in the synthesis did not all appear in the same branch, and with the list including predicted interactions [34] (Additional file 1).

To characterize the factors that may influence the distribution of the partners, we performed a Multiple Correspondence Analysis (MCA), taking into account the moment of appearance, the molecular weight of the ligand, the family, the kind of molecule (syntesized ligand, glycoprotein, etc.), the kind of signal (hormone, neuropeptide, etc.) and the function of the gene family (immunity, metabolism, etc.).

## Results

### Receptors and ligands are predominantly born in the same branch

**Fig. 3:**
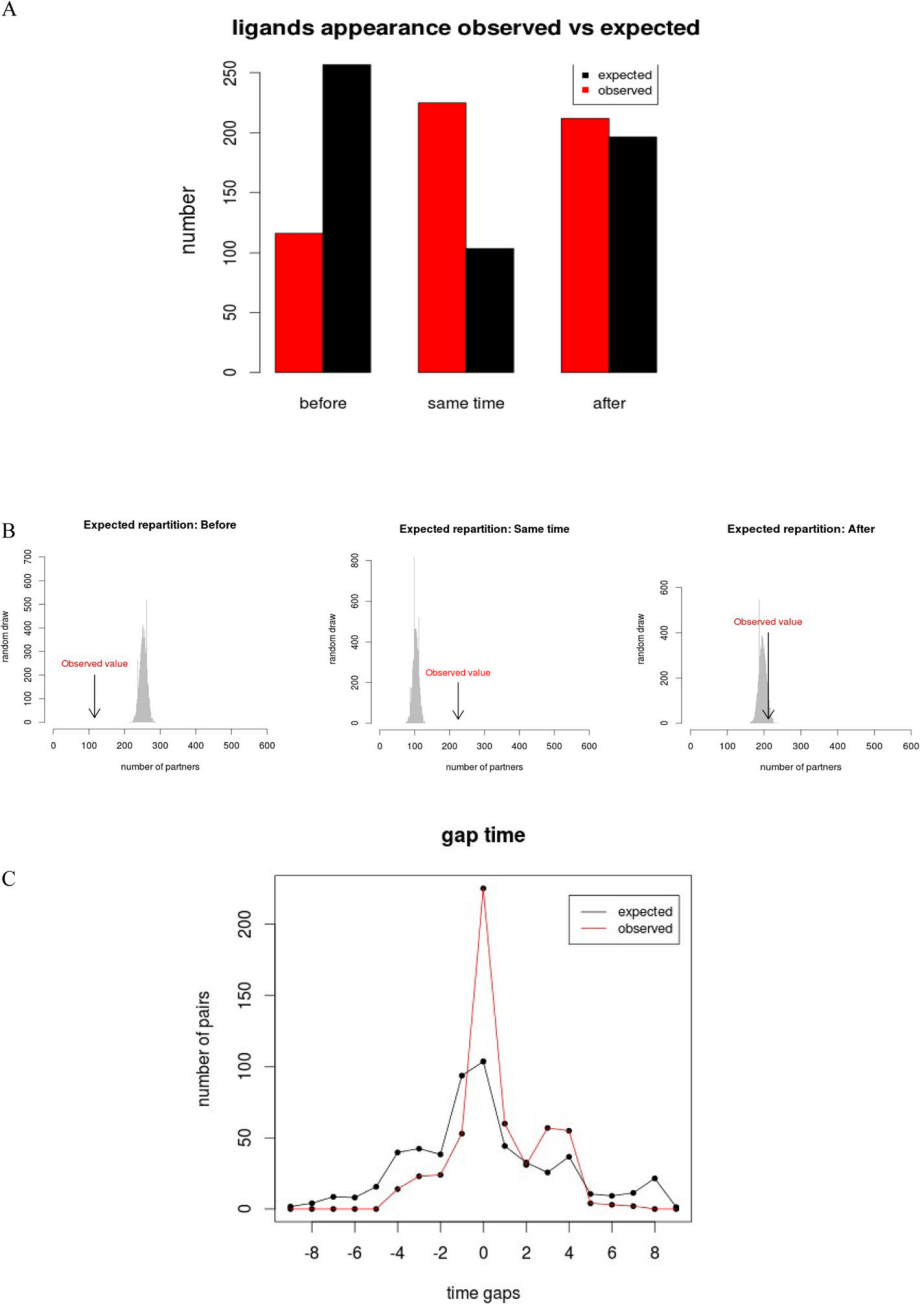
**a** Barplot of the global distribution of the 553 partners in each of the three categories. Category 1: Ligands which appeared before their receptors; Category 2: ligands which appeared in the same branch as their receptors; Category 3: ligands which appeared after their receptors. The x-axis represents the three categories, the y-axis represents the number of partners. The red bars correspond to the observed distribution. The black bars correspond to the expected distribution. 116 pairs were observed for which the first ligand of the considered receptor appeared before, against 256 expected. 225 pairs were observed for which the first ligand of the considered receptor appeared in the same branch, against 102 expected. 212 pairs were observed for which the first ligand of the considered receptor appeared after, against 195 expected. **b** Distribution of the 553 randomly selected partners, repeated 10,000 times (grey). The position of the observed number of partners is indicated with an arrow. **c** Distribution of the distance (in terms of branches of appearance) between all the genes encoding the ligands and their receptors. The red curve represents the observed data, and the black curve the expected distribution found with the random draws. 0 corresponds to a pair of ligand/receptor that appeared in the same branch. We observe an expected peak at 0 in the black curve due to the fact that a gap of 0 can be obtained over 10 branches, whereas a gap of 1 can only be obtained over 9 branches, a gap of 2 over 8 branches, etc. The negative values represent the genes encoding the ligand that appeared n branches before the gene encoding the receptor. The positive values correspond to the gene encoding receptors that appeared n branches before the gene encoding the ligands

Among the 553 pairs of ligand/receptor, we observed that the pairs were unequally distributed in the three categories. The number of pairs in LS (Ligands Synchronous) was not different from the number of pairs in the LA (Ligands After) category (40.69% vs 38.33%, *p*-value = 0.534, chi-square test), and the number of pairs in these two categories was higher than the number of pairs in the category LB (Ligand Before) (20.98%; *p*-value = 3.6e-09 LB vs LS, p-value = 1.2e-07 (LB vs LA) (Fig. 3a). Moreover, the majority (77/101) of ligands that result from a chain of synthesis were grouped in LB. The majority of the pairs found in LS appeared at the root of metazoa (branch 2, 48%), the root of vertebrates (branch 5, 16%) and the root of teleosts (branch 6, 9%).

We then evaluated the distribution of the partners against a theoretical distribution that assumes independence between protein appearance (Fig. 3a and b). Remarkably, we found that pairs where the receptor and the ligand appeared synchronously in the same branch (LS) is 2.5-fold higher than in the null distribution (*p*-value = 2.2e-16, chi-square test). In addition, for the pairs of ligand/receptor that did not appear at the same time, they appear in branches closer together than expected (Pearson correlation: *p*-value = 5.873e-05, *r* = 0.22), showing that pairs that do not appear in the same branch still tend to appear in neighboring branches (branch n-1 or n + 1) (Additional file 1). No such correlation was observed (Pearson p-value = 0.1363, *r* = − 0.071) for protein pairs with partners selected randomly according to observed branch frequencies Fb (see methods). Surprisingly, the observed number of human ligands that appeared before their receptors (LB) was 2-fold lower than the number expected from a null distribution (*p*-value = 3.6e-12, chi-square test). The observed number of human ligands that appeared after their receptor (LA) was not different from the number expected from the null distribution (*p*-value = 0.31).

The results are the same when we consider the list of 553 interactions with all the receptors, as well as with the list of 113 interactions with one member of each receptor’s family, and the lists without non peptide ligands and predicted interactions (Additional file 1).

### MCA analysis

**Fig. 4:**
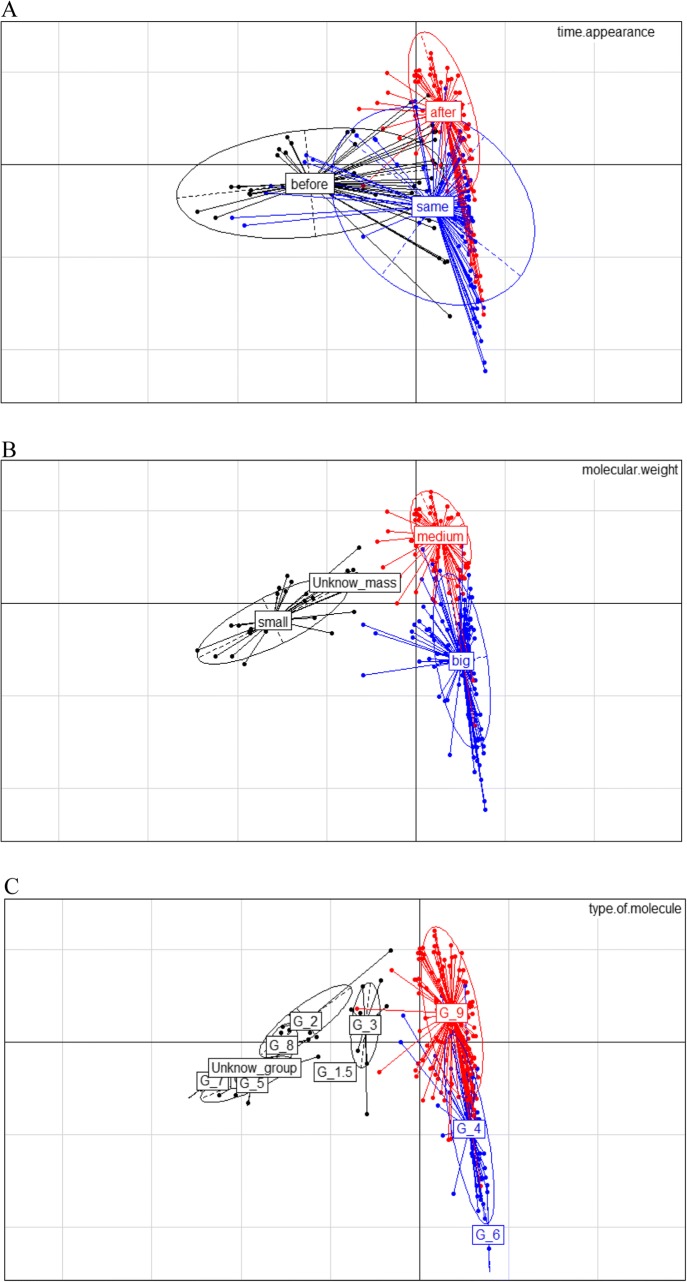
Spatial representation of the Multiple Correspondence Analysis. **a** Represents the pairs colored according to their categories of time of appearance (before in black, same branch in red, after in blue). **b** and **c** Represent the two traits which present the greatest influence on the results. **b** represents the distribution of the partners according to their molecular weight in dalton. Small: < 550 Da, Medium: 550 to 25,000 Da, Big: > 25,000 Da. **c** Represents the distribution of the type of molecule. Group 9 (red) are the “other proteins”, corresponding the free proteins as neuropeptides and not glycoprotein hormones, group 1, 1.5, 2, 3, 5, 7, 8 contain the amines, monoamines, catecholamines, lipids and derivatives, nucleotids and derivatives, the esters and the gaba. Group 4 contains the glycoproteins and group 6 (blue) the scleroproteins

Finally, a MCA analysis integrating 5 families of criteria identified two characteristics that were correlated with the moment of appearance of the ligand (Fig. 4a): the molecular weight of the ligand (Fig. 4b), and the type of molecules (see Additional file 1). We observed that the glycoproteins ligands are grouped all together in the MCA, and correspond to the same group of receptor-ligand pairs that appeared in the same branch. (Fig. 4c). We also observed in the MCA that the smallest ligands (< 550 Da) tend to appear before their receptor, the medium ligands (between 550 to 25,000 Da) tend to appear after, and the biggest ligands (more than 25,000 Da) tend to appear synchronously with their receptors. Moreover, we observed that glycoproteins tend to also appear simultaneously with their receptors, whereas the hormones and neuropeptides tend to appear after their receptor. Contrary to the co-evolution of the interaction that is influenced by the function of the partners [49], we did not observe any influence of the function of the interaction on the co-appearance of the receptor-ligand partners.

## Discussion

The fact that ligands appeared less frequently before their receptor than expected suggests that the birth of a ligand is more dependent on the prior existence of a receptor than the opposite, and that ligands are more likely to be replaced during evolution than receptors (21% of our distribution are ligands that appeared before their receptor, against 38% receptor that appeared before their ligand). For receptors whose the first ligand appeared before (Lb), we could hypothesize that the ligands interacted with receptors that were replaced by others during evolution, or that receptors evolved very quickly and that their branch of appearance is most ancestral than expected. In a recent study, it was demonstrated that membrane proteins, that include all our membrane receptors, evolve faster than free proteins [43]. In contrast, [44] suggested that receptor structures undergo a tighter constraint than the ligand, and that “receptors drive the evolution of ligands in invertebrates”. Our results seem in agreement with the latter hypothesis, which tends to suggest that the results of [43] might not affect all membrane proteins in the same way. A second hypothesis could be that these ligands were not “ligands molecules” until the receptor arrived.

Finally, there are several known cases of ligands binding to other molecules as well as to their membrane receptors. Such is the case of human albumin, ALB. Albumin is a ligand to receptors f-ALB in man [45] and FcR/CR in chickens [46]. However, serum albumin is also known for a variety of other functions or liaisons. Albumin binds water, as well as certain fatty acids, hormones, bilirubin and drugs (GeneCard [47, 48]). This seems to entail that part of the ligands which appeared without their receptors were selected for their function in other binding mechanisms.

Remarkably, the synchronous appearance of receptor and ligand pairs far exceeds expectations (2.5-fold more). This result shows that the birth of each partner in a receptor-ligand pair tends to be more synchronous than expected by chance. This discovery testifies to the dependence between two partners. The establishment of an interaction is largely favored by the fact that the two partners are present at the same time, the appearance of only one of them in a branch being not the dominant model. This suggests that many binding pairs did not change partners during evolution, and that both partners conserved their binding function since its moment of appearance. Our results confirm that the protein interactions are well-conserved during evolution, as previously shown [10, 49].

The number of branches that separate the moment of appearance of the receptor and its ligand was also determined, for the observed and randomized data (Fig. 3c). The number of pairs with distance 0 – corresponding to ligands and receptor that appeared in the same branch – is higher than the expected number, as previously shown, and the number of pairs for all the other distances (1–9) is almost always lower than the expected curve. However, unexpectedly, we also observed a peak in the observed curve for distances 3 and 4 (Fig. 3c). This peak of the curve corresponds to a group of 50 receptors (32 in peak 3 and 18 in peak 2) that appeared in Eumetazoa and Protostomians (branch 2 and 3), with their ligands appearing in Vertebrates and Teleosts (branches 5 or 6). Most are neuropeptides, with complex phylogenies that are difficult to reconstruct [50, 51]. For the pairs of this peak that were documented in the literature, most previous studies are in accordance with our timing of appearance for these proteins [51–60]. Nevertheless, three recent studies [61–63] focusing on kisspeptin, galanin, cholecystokinin, gastrin, neuromedin U, pyrokinin, sulfakinin and follicle stimulating hormone, obtained different results from ours and from those of other authors. In these three studies, the birth of the ligand was found to be older than expected by using only phylogeny, which would reassign 15 of pairs from LA to LS. These phylogenetic researches were conducted on few molecules, with methods that are still difficult to implement on the scale of a large dataset [51, 57, 61–63]. For those reasons, we believe that the number of pairs of ligand/receptor that appeared in the same branch is underestimated, and that the side peaks of the curve include partners that may have appeared in the same branch, although this may only be a small number.

The case of ligands resulting from a chain of biosynthesis is an exception. In our study, we have considered the ligand to be present if the enzymes necessary to its biosynthesis were too. Nevertheless, pathways involving alternatives enzymes in the biosynthesis process cannot be excluded, nor the fact that the biosynthesis pathway may have undergone alterations during evolution. This is the case of the mevalonate pathway which allows the conversion of acetyl-CoA into isopentenyl 5-diphosphat. This biosynthesis pathway was preserved across the animal world and can also be observed in bacteria. Three reactions occurs among phosphorylations involving ATP. The enzymes responsible for these reactions differ from one taxonomic group to the next. Specifically, the effects of a reorganisation can be observed between animals and bacteria with regards to enzyme folding [64]. Indeed, cases in which the ligand results from a biosynthesis chain should be treated with caution, due to a possible change of enzymes involved in the biosynthetic pathway.

We observed that many receptors appeared independently from their mammalian ligand. Interestingly, the fact that many ligands appear after their receptor was already observed [34]. In their study, these authors used phylostratigraphic approach to show that most ligands appear after their receptor. However, they did not consider the first ligand to have appeared, but rather investigated cases of coevolution of ligands once the first ligand and receptor have appeared. Furthermore, half of their interactions are predicted in silico, not experimentally determined, which add a lot of predicted ligands interacting with the same receptor. Moreover, a number of their interactions also involve G proteins that were removed from our study, because they are nor membrane receptors nor ligands.

### Relationship between the functional characteristics of the pair ligand-receptor and their moment of birth

The MCA analysis resulted in two significant factors that were correlated with the moment of appearance: the molecular weight of the ligand and the type of molecules (see Additional file 1). The glycoproteins ligands correspond to the same group of receptor-ligand pairs that appeared in the same branch. The smallest ligands (< 550 Da) tend to appear before their receptor, the medium ligands (between 550 to 25,000 Da) tend to appear after, and the biggest ligands (more than 25,000 Da) tend to appear synchronously with their receptors.

Because large proteins have more amino acids than small ones, they present more amino acids subjected to substitution than in small proteins. Moreover, for membrane anchored molecules, the amino acids at the surface of the molecules are more substituted than those present at the centre, the latter being the part that allows them to be implanted in the membrane [43]. One could hypothesize that when a ligand appears, a quick and localized succession of changes has a higher chance to give rise to a binding area (at the surface) than in small and not anchored ligands. Consequently, in such big molecules anchored in the membranes, the amino acids that will interact with a new receptor (that appeared in the same branch) may have more probability to appear by chance than in small molecules. If such an interaction presents a functional interest, the nascent binding pocket may rapidly be fixed in the branch of birth of the two partners.

### General remarks

A limit to our method was the difficulty to date the birth of small ligands that evolved quickly. Even after correcting the possible bias, we suspect that a small number of false positives are still present, but they are unlikely to change the main conclusions. Additional efforts in the development of phylogenetic tools and in the curation of genomic data may gradually help solve this problem. Furthermore, the increasing availability of new genome sequences, especially in branches currently under-represented in the tree of life, will allow a finer dating of receptor-ligand relative birth times. Another limit of our method was the large and different gaps of time that separate our different branches. Other studies could be redone using shorter time steps, on organisms that diverged more recently. In addition, it bears noting that the receptors or ligands which appeared before their current partner potentially have an as yet undiscovered current partner. In this regard, future studies may in time shed light on ligands and receptors interacting with known proteins, and whose time of appearance corresponds to one of the proteins in our list.

Moreover, to enrich our model, it would also be interesting to take into account other interacting molecules, including intracellular ligands. It has been shown, for example, that G-protein coupled receptors evolve faster in their extracellular portion than in the transmembrane and cytosolic regions [43, 65]. Finally, our study focused on membrane receptors and their ligands. Since it has been demonstrated that the evolution of the interaction was different between transient and stable complexes [66], the application of our methodology to other kinds of interaction should allow a finer dissection and modelling of the influence of interaction types on the evolutionary fates of the interacting partners.

## Conclusion

In the present study, we demonstrate that human ligands and their receptors appeared in the same evolutionary branches much more often than expected by chance, suggesting that when two binding molecules appear in a given branch, they are quickly submitted to purifying selection, which explains their conservation during evolution. This interdependence between the appearance of the membrane receptors and their ligands complements our knowledge of the evolution of binding partners, showing that before the well-studied co-evolution of the partners, we find a co-appearance scenario of these proteins. Thanks to the MCA, we observed that the biological function of the pairs of ligand receptors does not seem to play a role in the appearance of the interaction. However, the nature and the weight of the ligands were found to correlate with the moment of appearance, suggesting that the birth of the interaction is constrained by physical and chemical factors.

## Additional files

Additional file 1: Page 1 1: Branch of appearance of each of the 19,928 human genes. 2: Branch of appearance of the 1000 genes of bilaterians drawn randomly. Page 2: Branch of appearance of human genes for the mathematical model. Page 3: Branches of appearance of ligands and receptors. Page 4: List of receptor/ligand interactions. The file contains the interactions present in our database, as well as the interactions present in the list of Ramilowski et al., 2015. Page 5: List of ligands that have evolved rapidly. Page 6: List of characteristics of ligands and receptors used for MCA. The characteristics are the molecular weight before and after cleavage, the synthesis, the groups 1 to 9 according to the characteristics of the proteins (ex glycoproteins), the type of signal and the function of the interaction. (XLSX 578 kb)

Additional file 2: Part 1: List of Databases used to construct the receptor/ligand interaction Database. Part 2: Characteristics of the components of the MCA. Part 3: Explanation of the statistical model. Part 4 Organisms of the branches of the overall phylogenetic trees + organisms implemented by BLAST. Part 5: Supplementary discussion on the appearance times of ligands and receptors. Part 6: Results of the random statistical model for the different combinations used. Part 7: Explanation of the methodology to match the bases Ensembl and Ensembl metazoa. Part 8: Number of receptors according to their number of ligands. Part 9: List of ligands and receptors in our database. (ODT 46 kb)

## Abbreviations

LA: ligand after
LB: Ligand before
LS: ligand same time
MCA: Multiple correspondance analysis
