# Supplementary material for: Synchronous birth is a dominant pattern in receptor-ligand evolution
Source: BMC Genomics. 2018 Aug 14;19:611. doi: 10.1186/s12864-018-4977-2 (PMC6092800; doi:10.1186/s12864-018-4977-2)
Supplement: Supplementary file 1 [file 12864_2018_4977_MOESM2_ESM.odt]

Supplemental Data

1 Databases for the list

We extracted interacting partners from the DIP repository (“Database of Interacting Proteins”), which contains the official “Database of Ligand receptor partners”, (Graeber et al, 2001), (http://dip.doe-mbi.ucla.edu/dip/DLRP.cgi). We complemented this list with the receptome database ([http://www.receptome.org/](../../../../C:/h); http://hsuehlab.org/) and also integrated the Kyoto Encyclopedia of genes and genomes (http://www.genome.jp/kegg/brite.html), using the file: “Enzyme_linked receptors”, “Cytokines receptors”, “Ions Channels receptors”, “CAMs and their ligands”, and with the IUPHAR database (http://www.iuphar-db.org/), using the “G-protein-coupled receptors database”.

2 Multiple Correspondence Analysis (MCA)

Factor 1 Functions

Using the GeneCard data bank [http://www.genecards.org/](http://www.genecards.org/), we defined 15 main functions for the ligand.

1. Immunity

2. Homoeostasis

3. Reproduction and gestation

4. Embryonic development

5. Structure

6. Growth

7. Growth factor

8. Hematopoiesis and angiogenesis

9. Cell signalisation

10. Coagulation

11. Blood pressure

12. Neurotrophic factors

13. Nutrition

14. Metabolism

15. Circadian cycle

Factor 2 Molecular weight

In this category, we divided the ligands according to their molecular weight, as follows:

	-for molecules synthesised by one gene, we used the weight of the molecule before post translational modifications, using the website Uniprot [http://www.uniprot.org/](http://www.uniprot.org/)

	-for peptides resulting of cleavage of bigger molecules, we used the weight of the molecule before the cleavage.

	- for molecules that result from a chain of biosynthesis, the molecular weight was not available on Uniprot but could be found on specialised sites, on a case by case basis.

The molecular weights were divided into the following categories:

Lighter to 550 Da, comprised between 600 to 4,000 Da; between 4000 da and 10 kDa; between 10 and 25 kDa; between 30 and 100 kDa, and heavier than 100 kDa.

Factor 3 Receptor family

The receptors were divided according to their family. The super family was included in the MCA, whereas all the subfamilies were used to reproduce our test concerning their birth in the tree of life. In this case, we only considered the receptor that came first, along with its first ligand.

Factor 4 Families

The studied receptors are primarily known in the literature. We built the list of the gene families, and reduced the several genes that constitute a family within one family.

Super-family 1 : Ions channels receptors

-Cys-loop receptors

-ATP-gated channels

-Glutamate cationics receptors

Super-family 2 : GPCR

Class 1 :

-Hydroxytryptamine receptors

-Acetylcholine receptors

-Adenosine receptors

-Adrenoceptors

-Angiotensin receptors

-Apelin receptor

-Bile acid receptor

-Bombesin receptors

-Bradykinin receptors

-Cannabinoid receptors

-Chemerin receptor

-Chemokine receptors

-Cholecystokinin receptors

-Complement peptide receptors

-Dopamine receptors

-Endothelin receptors

-Formylpeptide receptors

-Free fatty acid receptors

-Galanin receptors

-Ghrelin receptor

-Glycoprotein hormone receptors

-Gonadotrophin-releasing hormone receptors

-GPR18, GPR55 and GPR119

-G protein-coupled estrogen receptor

-Histamine receptors

-Hydroxycarboxylic acid receptors

-Kisspeptin receptor

-Leukotriene receptors

-Lysophospholipid (LPA) receptors

-Lysophospholipid (S1P) receptors

-Melanin-concentrating hormone receptors

-Melanocortin receptors

-Melatonin receptors

-Motilin receptor

-Neuromedin U receptors

-Neuropeptide FF/neuropeptide AF receptors

-Neuropeptide S receptor

-Neuropeptide W/neuropeptide B receptors

-Neuropeptide Y receptors

-Neurotensin receptors

-Opioid receptors

-Orexin receptors

-Oxoglutarate receptor

-P2Y receptors

-Platelet-activating factor receptor

-Prokineticin receptors

-Prolactin-releasing peptide receptor

-Prostanoid receptors

-Proteinase-activated receptors

-QRFP receptor

-Relaxin family peptide receptors

-Somatostatin receptors

-Succinate receptor

-Tachykinin receptors

-Thyrotropin-releasing hormone receptors

-Trace amine receptor

-Urotensin receptor

-Vasopressin and oxytocin receptors

-Class A Orphans

class 2 :

-Calcitonin receptors

-Corticotropin-releasing factor receptors

-Glucagon receptor family

-Parathyroid hormone receptors

-VIP and PACAP receptors

class 3

-Calcium-sensing receptors

-GABAB receptors

-Metabotropic glutamate receptors

-Taste 1 receptors

Class FRIZZLED

-adhesion

Super-family 3 : enzyme receptors

class 1 : Receptor tyrosine kinases

-EGF receptor family

-Insulin receptor family

-PDGF receptor family

-FGF receptor family

-VEGF receptor family

-HGF receptor family

-TRK receptor family

-EPH receptor family

-AXL receptor family

-LTK receptor family

-TIE receptor family

-ROR receptor family

-DDR receptor family

-RET receptor family

-KLG receptor family

-RYK receptor family

-MuSK receptor family

classe 2 : Receptor serine/threonine kinases

-TGF-beta receptor type1

-TGF-beta receptor type2

Super-family 4: cytokine receptors

-hematopoietin family receptor

-interferon/IL-10 family receptor

-TNF receptor family

-OTHER..

Super-family 5 : Nuclear receptor, not taken into account in our study

Super-family 6 : CAM : cell adhesion molecules

Class 1 : Immunoglobulin superfamily

sub family 1 : natural killers

-two domains, long cytoplasmic tail

-two domains, short cytoplasmic tail

-three domains, long cytoplasmic tail

-three domains, short cytoplasmic tail

sub family 2 : leukocyte immunoglobulin-like receptor

-alpha

-beta

Class 2 : Integrin family

-integrin beta

-integrin alpha

sub family 3 : selectins

sub family 4 : LINK PROTEIN FAMILY

sub family 55 : CADHERINS

sub family 6 : Sialomucin Family

Super-family 7 : Cell surface proteoglycans

-Syndecan family

-Glypican family

-OTHERS

Super-family 8 : Lectin receptors

-NK-cell receptors

3 Models and Statistics

We compared the distribution of the pairs of ligand/receptor in the three categories (LB: the gene coding for the ligand appeared before the gene coding for the receptor, LS: the gene coding for the ligand appeared at the same time as the gene coding for the receptor, LA: the gene coding for the ligand appeared after the gene coding for the receptor) with a Chi2 test (H0: There is no difference in the distribution of pairs between the three categories). The distribution of the randomly selected pairs of genes was also tested with a Chi2 test (H0: There is no difference of distribution in the three categories between the pairs in our list and the pairs chosen randomly).

The average distance (in numbers of branches) between the time of appearance of the ligand and the time of appearance of the receptor was computed, and compared between the observed and the random pairs, using a Pearson correlation test, and a Linear regression. The simple linear model was performed (H0: there is no impact of the time of birth of the gene encoding the ligand on the time of birth on the gene encoding the receptor).

The difference between the percentages of conservation of the binding site and the percentages of conservation of all the amino acids of the sequence was tested using a Generalised Linear Model (GLM). The GLM that we used was a Poisson regression (the two H0 were respectively: The appearance of the ligand does not have any effect on the value of the percentage of conservation of the amino acids involved in the binding interface, and the amino acids involved in the binding interface are not more conserved than the other amino acids).

The representations of 3D structures of the proteins were built with Chimera (Pettersen al, 2004).

We used a Multiple Correspondence Analysis (MCA) to study which of the characteristics of the ligand has the greatest impact on the time of appearance. We defined 5 characteristics: receptor gene family, molecular weight, function, nature (peptide or not) and signal (see supplemental data). The AMC was performed using the R package ADE4 (Dray et al, 2007).

4 Organisms of the branches

| Mammalia | Dasypus novemcinctus (Armadillo); Otolemur garnettii (Bushbaby); Canis lupus familiaris (dog); Felis catus (cat); Bos taurus (caw): Ictidomys tridecemlineatus (squirel); Loxodonta africana (elephant); Nomascus leucogenys (gibbon); Cavia porcellus (guinea pig); Dipodomys ordii (kangaroo rat); Oryctolagus cuniculus (rabbit); Macaca mulatta (rhesus macaque); Callithrix jacchus ([New World monkeys](https://en.wikipedia.org/wiki/New_World_monkey)); Pteropus vampyrus (megabat); Myotis lucifugus (microbat), Mus musculus (mouse); Monodelphis domestica (opossum); Ailuropoda melanoleuca (panda); Ochotona princeps (pika); Sus scrofa (wild boar); Ornithorhynchus anatinus (platypus); Rattus norvegicus (rat); Sorex araneus (shrew); Tarsius syrichta (tarsier); Sarcophilus harrisii (tasmanian); Macropus eugenii (wallyby). |
| --- | --- |
| Reptilia | Alligator sinensis(Chinese alligator); camelus australis(South African ostrich); Coturnix japonica(Japanese quail); Gekko japonicus(Japanese gecko); Python bivittatus(Burmese python); Anas platyrhynchos(wild duck); Gallus gallus(chicken); Ficedula albicollis(collared flycatcher); Meleagris gallopavo(wild turkey); Taeniopygia guttata(zebra finch); Pelodiscus sinensis(Chinese softshell turtle); Anolis carolinensis(Carolina anole) |
| Amphibia | Ambystoma mexicanum(Mexican salamander); Cynops pyrrhogaster(Japanese fire); Hoplobatrachus rugulosus(frogs); Notophthalmus viridescens(eastern newt); Rana catesbeiana(American bullfrog); Rana pipiens(Northern Leopard Frog); Rhinella marina(cane toad); xenope laevis(African clawed frog); xenopus borealis(Marsabit clawed frog); xenopus laevis(Mwanza frog); xenopus tropicalis(western clawed frog); Taricha granulosa(rough-skinned newt) |
| Coelacanth | Latimeria chalumnae(cœlacanths); Lepidosiren paradoxa(South American lungfish); Protopterus_dolloi(African lungfishes); Lepidosiren paradoxa(South American lungfish); Neoceratodus forsteri(Queensland lungfish); Protopterus annectens(West African lungfish); Protopterus dolloi(African lungfishes) |
| Teleosteans | Astyanax mexicanus(cave fish); Poecilia formosa(Amazon molly); Gadus morhua(Atlantic cod); Takifugu rubripes(fugu); Oryzias latipes(Chinese rice fish); Xiphophorus maculatus(southern platyfish); Lepisosteus oculatus(spotted gar); Gasterosteus aculeatus(stickleback); Tetraodon nigroviridis(Tetraodon); Oreochromis niloticus(Tilapia); Danio rerio(Zebrafish) |
| Petromyzontidae | Petromyzon marinus(Lampreys); Lampetra fluviatilis(Lampreys); Lethenteron reissneri( Lampreys) |
| Cephalochordata | # Ciona intestinalis(Ciona); Ciona savignyi(Ciona); Botryllus schlosseri(golden star ascidian); Branchiostoma floridae(lancelet), Halocynthia roretzi(sea pineapple); Oikopleura Dioica(lancelets); Branchiostoma Lanceolatum(lancelets) |
| Protostomia | # Aedes aegypti(mosquito); Anopheles sinensis(mosquito); Aplysia californica(California sea hare); Bactrocera dorsalis(Bactrocera dorsalis); Biomphalaria glabrata(snail); Caenorhabditis remanei(nematode); Ceratitis capitata(fruitfly); Crassostrea gigas(oysters); Culex quinquefasciatus(mosquito); Drosophila ananassae(fruitfly); Drosophila biarmipes(fruitfly); Drosophila erecta(fruitfly); Drosophila eugracilis(fruitfly); Drosophila ficusphila(fruitfly); Drosophila persimilis(fruitfly); Drosophila rhopaloa(fruitfly); Drosophila simulans(fruitfly); Drosophila yakuba(fruitfly);Lingula anatina(Brachiopod); Loa loa(nematode); Lottia gigantea(sea snails); Lucilia cuprina(bottle fly); Musca domestica(housefly); oesophagostomum dentatum(nematodes) |
| Cnidaria; Ctenophora; Placozoa; Porifera | Acropora digitifera(coral); Acropora millepora(coral); Aiptasia pallida(cnidarian); Nematostella vectensis(anemone); Amphimedon queenslandica(sponge); Trichoplax adhaerens(Placozoa); Mnemiopsis leidyi(ctenophore) |
| Unicellular | Saccharomyces arboricola(Yeast); Saccharomyces cerevisiae(Yeast); Saccharomyces_eubayanus(Yeast). |

5 Description of the distribution

5 a. Ligands that appeared before their receptors: LB

First, we observed that 81 of the 116 ligands found in this category are smaller than 550 Da, and among them, 69 result from a chain of synthesis, and 2 (ATP and adenosine) are the smallest molecular compounds of the list.

Moreover, the receptors of this category often have the same ligand. Actually, there are 116 different receptors, for only 46 different ligands. This is the case of the Cholinergic Receptor Nicotinic family (CHRN), in which we found 10 receptors: CHRNA1, CHRNA2, CHRNA3, CHRNA4, CHRNA5, CHRNA6, CHRNA7, CHRNA8, CHRNA9, CHRNA10, CHRNB2, that appeared 580 my ago, and that all bind to acetylcholin, which appeared before. It is also the case for the Cholinergic Receptor Muscarinic family: CHRM, in which we find 5 receptors, CHRM1, CHRM2, CHRM3, CHRM4, CHRM5, which appeared 713 my ago, and that also bind to acetylcholin. Overall, there are 15 different receptors, belonging to two close families (the same superfamily), which bind to the same ligand. Both families appeared after the ligand. For the first family that appeared (CHRM), the interaction would have taken place immediately in the branch in which the receptor appeared, because the large diversification of 10 receptors took place in the same branch, and the two families diverged at only one branch of interval.

The category LB also comprises the most ancient ligands. Actually, 60 interactions involve a ligand whose coding gene appeared in unicellular organisms (1500 my ago) and that was conserved until humans, like ATP, adenosin, glutamate, acetylcholine or tryptophan. On the other hand, we found that the gene coding for the human receptor of these ligands appeared either in the first metazoa (713 my, like Glycine Receptor: GLRA1-4; GRID1-2; GR1K4-5; GRIN1, 2A, 3A, 2B; Adenosine Receptor: ADORA1, 2, 3; Melatonin Receptor: MTNR1A,B) etc. or in protostomians (-580 my) (Hydroxytryptamine Receptor: HTR1A-E; ADRA1A,B,D; Cholinergic Receptor Nicotinic: CHRNA1-6) etc.

The most plausible hypothesis is that the unicellular organism and the plants (that we did not take into account in our study) have other receptors which may not be phylogenetically related to the human ones, and that a replacement occurred in the animal lineage.

We also found some pairs of ligand/receptors whose partners appeared with 3,4 or more branches of distance. It is the case for the Nerve Growth Factor Receptor NGFRAP1, that was found to appear in mammals, whereas its first ligand appeared in vertebrates. In this case, a further study would be interesting.

5 b. Ligands that appeared at the same time as their receptors: LS

In this category, the couples belong to the 15 systems defined for the AMC. However, a large part of the molecules of the structure, adhesion/differentiation and reproduction were found in this category. More than half of the interactions present in LS appeared in branch 2 (First metazoa) and 3 (Protostomians). The results of the AMC also showed that the ligands that are glycoproteins mostly appeared simultaneously. Many of the glytoproteins ligands are inserted in the extracellular matrix, and represent the biggest molecules of our dataset (> 30 kDa). We can therefore establish a profile for some of the ligands whose probability to appear at the same time as their receptor is high: big glycoproteins (>30kDa) inserted in the matrix or in the membrane. For example, we find Integrin receptors (ITGB1, 2, 3, 4, 5, 6, 8; ITGA1, 2, 3, 5, 6, 7, 8, 9, 10, 11), Frizzled Class Receptors with their ligands, the Wnt Family Member. There is also the Fibroblast Growth Factor Receptors, with their ligands the Fibroblast Growth Factors.

In this category, we also find ligands which have resulted from a chain of synthesis, and for which the several genes coding for the enzymes involved in the synthesis appeared at the same time. It is the case for the genes involved in the biosynthesis of the serotonine and the dopamine, that is genes encoding the tryptophan hydroxylase, the hydroxytryptophan decarboxylase, the dopa decarboxylase, and the monoamine oxydase, which appeared at the same time as genes coding for the Hydroxytryptamine Receptor (HTR2A, 2A,2C, 4, 6), and Adrenoceptor (ADRB1, 2, 3).

5 c: Ligands that appeared after their receptor: LA

In this category, we draw a first distinction between the partners that appeared only one branch apart, and the partners that appeared further in time.

Concerning the partners that appeared only one branch apart, although distributed inside all the branches, we find 45 cases for which the receptor appeared in branch 5, including the lamprey (~550 ma), and the ligand at branch 6, excluding the lamprey (~420 ma), which was confirmed by the synteny. Among these couples, 37 belong to the immune system, and 8 to other families (Opioid Growth Factor Receptor OGFR; Kininogen receptor: GPR135; GDNF Family Receptor: GFRA3, GFRA2, GFRA1, GFRA4; Prolactin Receptor: PRLR; Growth Hormone Receptor: GHR).

As stated in the paper, there is a group of about 85 couples for which the receptor appeared in the first metazoas (branch 2) or in the protostomians (branch 3), and for which the first ligand appeared in the vertebrates (branch 5 or branch 6). In these cases, the AMC showed that the majority are neuropeptides, with a medium weight (between 4000 and 25 kDa). Among these, we can find the adrenomedullin, the angiotensin, the neuromedin, the kininogens, the cholecystokinin, the galanin, the motilin, etc.

These ligands are almost neuropeptides and are involved in a lot of functions of the organisms, so few specialized. Ligands that appeared after their receptor, that is to say more recently, could justify that they are less specialised than more ancestral ligands that had more time to specialise according to their receptors, and to acquire a specific function. The fact that these molecules are multifunctional could explain why these ligands are more recent and so less specialised that more ancestral molecules.

Ultimately, we observed that the receptors belonging to LA come from gene families smaller than those of the 2 previous categories. This would suggest that the diversification allowed by more recent ligands was not as important as the diversification of the receptors in the other two categories, in which a more ancestral appearance of the ligand gave the receptors families enough time to diversify widely.

6 Alternative models of distribution

The distribution of the partners was also observed:

- After removing the 52 pairs for which the ligand resulted from a chain of synthesis, the process of which involved enzymes that did not appear in the same branch

- After grouping the pairs according to the family of their receptor. For each family of receptors, we only recovered the first receptor to have appeared, along with its first ligand.

- By implementing our list with a list of predicted interactions. When reducing this new list by using only the first ligand of each receptor, we found that only 27 interactions in the LB and LS behaved differently. This did not impact the results.

We observed the same results when the pairs were grouped by families of receptor genes (LS: p-value = 5.6e-05; LB: p-value = 2.9e-4; LA: p-value = 0.73), and when we removed the ligands which resulted from a chain of synthesis but for which the enzymes involved in the synthesis did not appear in the same branch. However, in this case, the number of ligands that appeared before their receptor (LB) was even smaller than when they were not removed from the list (LB: p-value = 2.2e-16; LS: p-value = 6.74e-16; LA: p-value = 0.69).

To make sure that the ligands whose coding genes evolved quickly did not affect our data, we repeated our experiments after removing them.

In order to do this, the human sequence, and the sequence of one of the species who branch at the root of the tree, were selected for each ligand. They were aligned using local alignment. The scores of alignments were recovered and ploted. For each brach, the ligands  with the weakest  alignment scores were removed. Weak scores correspond to the bottom bar of the boxplot. We removed 79 ligands (see excel file). The repartition of the molecules was not affected by this operation.

7: Correspondance of the 2 databases of Ensembl and Ensembl Metazoa

In order to assess the distribution of the 19982 human genes present in Ensembl, the roots of each tree were determined. We found the following proportions:

To estimate the correspondance between Ensembl and Ensembl Metazoa, we had to determine what proportion of the trees that rooted in branch 3 (bilaterians) actually rooted in branch 2 (first metazoas).

To do this, we randomly selected 1/10 of the 7603 genes that rooted in  branch 3, and looked which proportion of them finaly rooted in the branch 2.

8: Number of receptors having one to more than 10 ligands

|  | 1 ligand | 2 ligands | 3 ligands | 4 ligands | 5 ligands | 6 to 10 ligands | more than 10 ligands |
| --- | --- | --- | --- | --- | --- | --- | --- |
| Number of receptors | 250 | 125 | 60 | 42 | 15 | 36 | 15 |

9: List of receptors/ligands

IL3RA	IL3

GABBR1	GAD1

GABBR2	GAD1

IL1RAPL1	IL18

LPHN2	SHANK1

LPHN3	FLRT2

LPHN3	FLRT3

IL11RA	IL11

CCR9	CCL25

LILRB1	HLA-G

LILRB1	HLA-A

LILRB1	HLA-B

LILRB1	HLA-C

LILRB1	HLA-F

LILRB1	B2M

OGFR	Penk

MCHR2	PMCH

MC3R	POMC

MC3R	AGRP

VIPR1	VIP

VIPR1	ADCYAP1

GLP1R	GCG

CRHR1	CRH

CRHR1	UCN

CRHR1	UCN3

CCKBR	CCK

CCKBR	GAST

AGTR2	AGT

DCC	NTN1

DCC	NTN4

NMUR2	NMU

PROKR1	PROK1

EPHA8	EFNA5

EPHA8	EFNA3

EPHA8	EFNA2

EPHA8	EFNA1

EPHA8	EFNA4

GFRA3	ARTN

GFRA2	GDNF

GFRA2	NTRN

PROCR	PROC

F3	F7

HRH4	HDC

NGFRAP1	BDNF

NGFRAP1	NTF5

ADRB2	DBH

ADRB2	PNMT

GPR26	TPH1

BAMBI	INHBA

BAMBI	INHBB

GPC1	slit2

GPC1	SLIT3

GPC1	SLIT1

GPC1	COL4

ROBO4	slit2

ROBO2	slit2

EPHB4	EFNB2

EPHB4	EFNB1

EPHB4	EFNB3

GPR119	DBH

GPR119	OEA

GPR119	LPC

DRD5	TH

DRD5	DDC

HRH2	HDC

PAQR3	CYP11A1

NCSTN	PSEN1

NCSTN	PSEN2

NCSTN	PSENEN

FGFR1	FGF1

FGFR1	FGF4

FGFR1	FGF8

FGFR1	FGF3:

FGFR1	FGF23

FGFR1	FGF5

FGFR1	FGF2

FGFR1	FGF6

FGFR1	FGF9

FGFR1	FGF10

FGFR1	FGF11

FGFR1	FGF12

FGFR1	fgf12b

FGFR1	fgf13

FGFR1	fgf14

FGFR1	fgf16

FGFR1	fgf17

FGFR1	fgf18

FGFR1	fgf19

VIPR2	VEGFC

VIPR2	VIP

VIPR2	ADCYAP1

ITGA11	COL1A1

BDKRB1	KNG1

PROKR2	PROK2

PROKR2	PROK1

ATP6AP2	REN

KLRK1	ULBP2

KLRK1	ULBP3

TNFRSF14	TNFSF14

TNFRSF14	LTA

TNFRSF14	TNFSF1

DARC (ouACKR1)	CXCL1

DARC (ouACKR1)	other ccl

HTR2A	TPH1

HTR1F	TPH2

HTR7	TPH3

HTR4	TPH4

HTR5A	TPH5

ADRA2C	DBH

ADRA2C	PNMT

ADRA1A	DBH

ADRA1A	PNMT

ADRA2A	DBH

ADRA2A	PNMT

GPR161	DBH

AMFR	GPI

GFRA1	GDNF

GFRA1	NTRN

GPR135	KNG1

HMMR	HAS2

LEPR	LEP

RXFP4	RLN3

RXFP4	INSL5

RXFP4	kng1

IGF2R	IGF2

IGF2R	GZMB

IL2RA	IL2

IL2RB	IL2

IL2RB	IL15

IL2RG	IL7

IL2RG	IL9

IL2RG	IL4

IL2RG	IL2

IL2RG	IL15

IL2RG	IL21

IL2RG	IL13

IL7R	IL7

IL7R	TSLP?

IL15RA	IL15

ITGA6	DUSP18

ITGA6	LAMB1

ITGA6	LAMA1

ITGA6	LAMA2

ITGA6	LAMB3

ITGA6	LAMC2

ITGA6	LAMA3

ITGA6	LAMA4

ITGA6	LAMA5

ITGA6	LAMB2

ITGA6	ADAM9

ITGA6	ADAM2

ITGA1	DUSP18

ITGA1	LAMB1

ITGA1	LAMA2

ITGA1	COL4A1

ITGA2	DUSP18

ITGA2	LAMB1

ITGA2	LAMA1

ITGA2	CHAD

ITGA2	COL1A1

ITGA2	COL2A1

ITGA2	FN1

ITGA2	ICAM4

ITGAD	VCAM1

ITGAD	ICAM3

ITGAE	CDH1

ITGAL	ICAM1

ITGAL	ICAM5

ITGAL	ICAM4

ITGAL	ICAM3

ITGAL	ICAM2

ITGAL	LFA-1

ITGAM	CTGF

ITGAM	CYR61

ITGAM	ICAM4

ITGAM	F10

ITGAM	c3b

ITGAM	ICAM1

ITGAM	FGA

ITGAM	ICAM2

ITGAM	CR3

ITGAX	C3

ITGAX	FGA

ITGAX	ICAM1

ITGAX	ICAM4

ITGB3	ADAM15

ITGB3	ADAM23

ITGB3	CYR61

ITGB3	FN1

ITGB3	VTN

ITGB3	ICAM4

ITGB4	DUSP18

ITGB4	LAMB1

ITGB4	LAMA1

ITGB4	LAMA2

ITGB4	LAMB3

ITGB4	LAMC2

ITGB4	LAMA3

ITGB5	ADAM9

ITGB5	FN1

ITGB5	TGFB1

ITGB5	TGFB2

ITGB5	TGFB3

ITGB5	TGFBI

ITGB5	TGFBN

KLRC1	HLA-E

KLRC1	HLA-G

KLRC2	HLA-E

KLRD1	HLA-E

KLRD1	HLA-G

RPSA	DUSP18

RPSA	LAMA1

RPSA	LAMA2

RPSA	LAMB2

RPSA	PRND

MPL	THPO

ADCYAP1R1	ADCYAP1

ADRA1D	DBH

ADRA1D	PNMT

ADRA1D	DDC et TH 

ADRA1B	DBH

ADRA1B	PNMT

ADRA1B	DDC et TH 

ADRA2B	DBH

ADRA2B	PNMT

ADRA2B	DDC et TH 

AGTR1	AGT

AVPR1A	AVP

C5AR1	C5

CALCR	CALCB

CALCR	CALCA

CALCR	IAPP

CALCR	CCK

CCKAR	CCK

CCKAR	GAST

CCR1	CCL6

CCR1	CCL2

CCR1	CCL3

CCR1	CCL14

CCR1	CCL7

CCR1	CCL15

CCR1	CCL16

CCR1	CCL23

CCR1	CCL5

CCR1	CCL13

CCR2	CCL2

CCR2	CCL7

CCR2	CCL12

CCR2	CCL13

CCR2	CCL16

CCR2	CCL8

CCR2	CCL13

CCR3	CCL5

CCR3	CCL7

CCR3	CCL8

CCR3	CCL11

CCR3	CCL15

CCR3	CCL24

CCR3	CCL26

CCR3	CCL28

CCR3	CCL13

CCR5	CCL3

CCR5	CCL4

CCR5	CCL5

CCR5	CCL8

CCR5	CCL14

CCR5	CCL11

CCR5	ccl7

CCR5	CCL13

CCR7	CCL19

CCR7	CCL21

CHRM1	CHAT

CHRM2	CHAT

CHRM3	CHAT

CHRM4	CHAT

CNTFR	np

CNTFR	CNTF

CNTFR	CLCF1

CRHR2	CRH

CRHR2	UCN

CRHR2	UCN3

CRHR2	UCN2

CX3CR1	CX3CL1

CXCR4	CXCL12

DRD1	TH

DRD1	DDC

DRD2	TH

DRD2	DDC

DRD3	COMT

DRD3	TH

DRD3	DDC

DRD4	TH

DRD4	DDC

EDNRA	EDN1

EDNRA	EDN2

F2R	F2

F2RL3	F2

F2RL3	PRSS1

FPR1	ANXA1

FPR1	fMLP

FPRL1	CAMP

FSHR	CGA

FSHR	FSHB

GALR1	GAL

GALR1	GALP

GALR3	GAL

GALR3	GALP

GCGR	GCG

GHRHR	GHRH

GIPR	GIP

GNRHR	GNRH1

MLNR	MLN

CXCR3	CXCL9

CXCR3	CXCL10

CXCR3	CXCL11

CXCR3	CXCL12

CXCR3	CCL21

GRM4	GAD1

HRH1	HDC

HTR1A	TPH1

HTR1A	DDC

HTR1B	TPH1

HTR1B	DDC

HTR1D	TPH1

HTR1D	DDC

HTR1E	TPH1

HTR1E	DDC

HTR2B	TPH1

HTR2B	DDC

HTR2C	TPH1

HTR2C	DDC

HTR6	TPH1

HTR6	DDC

IL8RA (or cxcr1)	CXCL6

IL8RA (or cxcr1)	IL8

IL8RB	CXCL2

IL8RB	CXCL3

IL8RB	CXCL5

IL8RB	CXCL6

IL8RB	PPBP

IL8RB	IL8

IL8RB	CXCL1

LRP5	APOE

LRP5	WNT7B

LRP5	SOST

LTBR	TNFSF14

LTBR	LTB

LTBR	LTA

LTBR	TNF

MAS1	AGT

MC2R	POMC

NMBR	NMB

NPY1R	PYY2

NPY1R	NPY

NPY1R	PYY

NPY2R	NPY

NPY2R	PYY

NTSR1	NTS

OPRK1	POMC

OPRK1	PDYN

OPRL1	PNOC

OPRM1	POMC

OXTR	OXT

PTGFR	PTGS2

PTHR1	PTHLH

SCTR	SCT

TACR1	TAC1

TACR1	TAC4

TACR1	TAC3

TACR2	TAC1

TACR2	TAC3

TNFRSF7	TNFSF7

TNFRSF7	CD5

TRHR	GPHA2

TRHR	GPHB5

TRHR	CGA

TRHR	TSHB

TRHR	TRH

TSHR	GPHB5

TSHR	TSHB

ADRB1	DBH

ADRB1	PNMT

ADRB1	DDC and TH 

ADRB3	DBH

ADRB3	PNMT

ADRB3	DDC and TH 

AVPR2	AVP

BDKRB2	KNG1

EDNRB	EDN3

EDNRB	EDN1

EDNRB	EDN2

HCRTR1	HCRT

HCRTR2	HCRT

LHCGR	LHB

LHCGR	CGA

LHCGR	CGB

SSTR1	SST

SSTR1	CORT

SSTR2	SST

SSTR2	CORT

SSTR3	CORT

SSTR3	SST

SSTR4	CORT

SSTR4	SST

SSTR5	CORT

SSTR5	SST

NEO1	RGMA

NEO1	NTN2L

C3AR1	C3

C3AR1	C3a 

CCR6	CCL20

CMKLR1	RARRES2

F2RL2	F2

GHSR	GHRL

GHSR	CORT

GLP2R	GCG

PRLHR	PRLH

NPFFR2	NPFF

NPFFR2	NPAF 

PTHR2	PTH

PTHR2	TIP39

AGTRL1	APLN

CCR8	CCL1

CCR8	SCYA4

CCR8	SCYA17

GPR23	LIPH

GPR23	ENPP2 

XCR1	XCL1

XCR1	CXCL2

NPBWR1	NPW

NPBWR1	NPB

NPBWR2	NPW

NPBWR2	NPB

GRPR	NMB

GRPR	GRP

CALCRL	ADM

CALCRL	CALCA

CALCRL	CALCB

CALCRL	ADM2

IL21R	IL21

CDON	NTN2L

CDON	SHH

ADIPOR1	ADIPOQ

MC4R	AGRP

MC4R	POMC ?

MC5R	POMC

MTNR1A	AANAT

MTNR1A	ASMT

MTNR1B	AANAT

MTNR1B	ASMT

PPYR1	PYY

PPYR1	NPY

PPYR1	PPY

ARTS-1	Nucb2

ARTS-1	HLA

PLAUR	PLAU

IL20RB	IL20

IL20RB	IL24

IL20RB	IL19

NPY5R	PYY

NPY5R	NPY

AVPR1B	AVP

IL17RB	IL25

IL17RB	IL17B

IL17RB	IL17E

PTGDR	PTGDS

CXCR6	CXCL16

LRRC4C	NTNG1

LRRC4C	NTNG2

PTPRF	NID1

PTPRS	HSPG2

PTPRS	PPFIA1 ?

EPHA6	EFNA4

EPHA6	EFNA5

EPHA6	EFNA2

EPHA6	EFNA3

EPHA6	EFNA1

ADMR	ADM

HRH3	HDC

PLXNB1	SEMA4D

PLXNA4	SEMA3A

PLXNA4	SEMA6

PLXND1	SEMA3E

PLXND1	SEMA4A

PLXND1	SEMA3C

PLXND1	SEMA3A

HHIP	IHH

HHIP	SHH

HHIP	DHH

SCARF1	CALR

SORT1	NGFB

SORT1	BDNF

SDC1	SLIT1

SDC1	SLIT2

SDC1	THBS1

SDC2	FN1

you	FGF2

SDC4	FGF2

SDC4	ADAM12

GFRA4	PSPN

ROBO3	NCAM1

ROBO3	SLIT1

ROBO3	NCAM2

SORL1	APOE

NTSR2	NTS

TGFBR3	INHA

TGFBR3	TGFB2

TGFBR3	TGFB1

TGFBR3	TGFB3

CHRM5	CHAT

TRAF2	GSTP1

LPHN1	SHANK1

LPHN1	TENM2

TACR3	TAC1

CCR10	CCL28

CCR10	CCL27

CCR10	CCL4

CCR10	CCL2

CCR10	CCL8

RXFP3	RLN3

LRP8	APOE

LRP8	RELN

ADIPOR2	ADIPOQ

F2RL1	F2

F2RL1	ENTK

GPR103	P518

ATRN	ASIP

LRRN6A	OMG

LRRN6A	RTN4

LRP11	LTF

ITGA10	COL2A1

TNFRSF18	TNFSF18

GALR2	GAL

GALR2	GALP

PRLR	PRL

GPR77	C5

BOC	SHH

BOC	IHH

BOC	DHH

NRXN3	NXPH1

NRXN3	NXPH3

NRXN3	NLGN1

NRXN3	NLGN2

NRXN3	NLGN3

NRXN1	NXPH1

NRXN1	NXPH3

NRXN1	NLGN1

NRXN1	NLGN2

NRXN1	NLGN3

NRXN2	NXPH1

NRXN2	NXPH3

NRXN2	NLGN2

NRXN2	NLGN3

HTR5B	TPH1

CD36	THBS1

SCARB1	PTDSS1

SCARB1	APOE

SCARB1	APOA2

SCARB1	APOA1

UTS2R	UTS2

UTS2R	UTS2B

CD44	HAS2

CD44	SPP1

CD44	COL1A1

CD44	COL4A

CD44	FN1

CD44	LGALS9

CD47	THBS1

CD47	THBS2

SIRPA	CD47

SDC3	COL5A3

ALK	MDK

ALK	PTN

FZD9	WNT1

FZD9	WNT2

FZD9	WNT7

TNFRSF10A	TNFSF10

BMPR1B	GDF5

BMPR1B	BMP7

BMPR1B	BMP2

BMPR1B	BMP4

BMPR1B	BMP15

BMPR1B	TNFSF11

BMPR1B	BMP3

BMPR1B	BMP6

BMPR1B	BMP8

BMPR1B	BMP10

TNFRSF11B	TNFSF11

TNFRSF11B	TNFSF10

TNFRSF11B	TNFSF13

TNFRSF10B	TNFSF10

NRP1	SEMA3A

NRP1	VEGF

NRP1	PGF

NRP1	L1CAM

NRP1	SEMA3B

NRP1	VEGFB

TNFRSF10C	TNFSF10

TNFRSF13B	TNFSF13

TNFRSF13B	TNFSF12

TNFRSF13B	TNFSF13B

IL1RAP	IL1B

PLXNB2	SEMA4D

PLXNB2	SEMA4C

PLXNB2	SEMA4G

MUSK	AGRIN

EPHB6	EFNB1

EPHB6	EFNB2

EPHB6	EFNB3

FZD6	WNT4

NRP2	VEGFC

NRP2	SEMA3F

NRP2	SEMA3C

NRP2	FIGF

NRP2	VEGF

PLXNC1	SEMA7A

RAMP1	CALCB

RAMP1	ADM2

RAMP1	CALCA

RAMP2	ADM

RAMP2	CALCA

RAMP2	IAPP

CALCRL	WNT5A

CALCRL	WNT11

CALCRL	CALCA

CALCRL	CALCB

CALCRL	WNT2B

TNFRSF21	TNF

LRP6	DKK1

LRP6	SOST

LRP6	CTGF

LRP6	DKK2

LRP6	WNT3

LRP6	RSPO1

LRP6	wnt1

NTRK3	NTF3

NTRK3	NTF5

NTRK3	NTF4

UNC5C	NTN1

UNC5C	robo4

TNFRSF6B	TNFSF15

TNFRSF6B	FASLG

TNFRSF6B	TNFSF14

TNFRSF6B	TNFSF6

EGFR	EGF

EGFR	EREG

EGFR	AREG

EGFR	BTC

EGFR	HBEGF

EGFR	TGFA

LDLR	APOE

LDLR	APOB

LDLR	PF4

LDLR	APOH

TFRC	TF

ERBB2	NRG1

ERBB2	ACPP ?

NTRK1	NGFB

NTRK1	NTF5

NTRK1	NTF3

ITGB1	FN1

ITGB1	RELN

ITGB1	VCAM1

ITGB1	LAMA1

ITGB1	LAMA2

ITGB1	DUSP18

ITGB1	LAMB1

ITGB1	LAMB3

ITGB1	LAMC2

ITGB1	LAMA3

ITGB1	LAMA4

ITGB1	LAMA5

ITGB1	LAMB2

ITGB1	PLG

ITGB1	ADAM17

ITGB1	ADAM15

ITGB1	ADAM9

ITGB1	ADAM12

ITGB1	ADAM2

ITGB1	COL2A1

ITGB1	COL1A1

ITGB1	CHAD

ITGB1	FN1

ITGB1	COL4A1

ITGB1	NPNT

ITGB1	COL18A1

INSR	INS

INSR	PBEF1

INSR	IGF2

ITGAV	ADAM9

ITGAV	SPP1

ITGAV	ICAM4

CSF1R	CSF1

CSF1R	il34

RET	NRTN

RET	GDNF

RET	PSPN

RET	ARTN

IGF1R	IGF1

IGF1R	IGF2

IGF1R	INS

NGFR	NGFB

NGFR	NTF3

NGFR	NTF5

NGFR	BDNF

NGFR	NTF4

NGFR	NGF

ITGA2B	FN1

ITGA2B	ICAM4

ITGA2B	BDNF

MET	SEMA4D

MET	HGF

MET	SEMA5A

ITGA5	FN1

ITGA5	ADAM17

ITGA5	ADAM15

ITGA5	ADAM23

ITGA5	CYR61

ITGA5	VTN

ITGA5	COL18A1

IL6R	IL6

PDGFRB	PDGFD

PDGFRB	PDGFB

PDGFRB	PDGFA

KIT	KITLG

KIT	SCGF

ITGA4	FN1

ITGA4	VCAM1

ITGA4	MADCAM1

SELL	CFH

SELL	CD34

SELL	PODXL2

IL1R1	IL1A

IL1R1	IL1B

IFNGR1	IFNG

CSF2RA	CSF2

NPR1	NPPA

NPR1	NPPB

SELP	SELPLG

SELP	CD24

SELP	PODXL2

PDGFRA	PDGFA

PDGFRA	PDGFB

PDGFRA	PDGFC

SELE	SELPLG

SELE	ITGAM

SELE	ITGB2

SELE	CD44

SELE	PODXL2

SELE	GLG1

IFNAR1	IFNA1

IFNAR1	IFNW1

IFNAR1	IFNB1

IFNAR1	IFNA4

IFNAR1	IFNA5

IFNAR1	IFNA16

IFNAR1	IFNA8

IFNAR1	IFNA14

IFNAR1	IFNA21

IFNAR1	IFNA2

IFNAR1	IFNA13

IFNAR1	IFNA6

IFNAR1	IFNA7

IFNAR1	IFNA10

IFNAR1	IFNA17

NPR3	NPPA

NPR3	NPPB

NPR3	NPPC

FLT1	VEGF

FLT1	VEGFB

FLT1	PGF

FLT1	VEGFA

ITGB6	FN1

ITGB6	FBN1 ?

ITGB6	TNC ?

ITGB6	TGFB1

ITGB6	TGFB2

ITGB6	TGFB3

ITGB6	TGFBN

EPOR	EPO

TNFRSF1A	TNF

TNFRSF1A	LTA

TNFRSF1B	OMG

TNFRSF1B	TNF

TNFRSF1B	LTA

NPR2	NPPC

NPR2	NPPB

NPR2	NPPA

EDG1	ACP6

EDG1	shingosine !

EPHA1	EFNA1

EPHA1	EFNA2

EPHA1	EFNA3

EPHA1	EFNA4

EPHA1	EFNA5

FGFR2	FGF23

FGFR2	FGF2

FGFR2	FGF4

FGFR2	FGF9

FGFR2	FGF10

FGFR2	PF4

FGFR2	FGF5

FGFR2	FGF8

FGFR2	FGF7

FGFR2	FGF22

FGFR2	FGF3

FGFR2	FGF1

FGFR2	FGF6

FGFR2	FGF11

FGFR2	FGF12

FGFR2	FGF12B

FGFR2	FGF13

FGFR2	FGF14

FGFR2	FGF16

FGFR2	FGF17

FGFR2	FGF18

FGFR2	FGF19

ERBB3	NRG2

ERBB3	NRG1

ERBB3	BTC

FGFR4	FGF5

FGFR4	FGF4

FGFR4	FGF8

FGFR4	FGF2

FGFR4	FGF1

FGFR4	FGF19

FGFR4	FGF23

FGFR4	FGF6

FGFR4	FGF9

FGFR4	FGF11

FGFR4	FGF12

FGFR4	FGF12B

FGFR4	FGF13

FGFR4	FGF14

FGFR4	FGF16

FGFR4	FGF17

FGFR4	FGF18

FGFR3	FGF2

FGFR3	FGF8

FGFR3	FGF4

FGFR3	FGF8

FGFR3	FGF9

FGFR3	FGF18

FGFR3	FGF1

FGFR3	FGF11

FGFR3	FGF12

FGFR3	FGF12B

FGFR3	FGF13

FGFR3	FGF14

FGFR3	FGF16

FGFR3	FGF17

FGFR3	FGF18

FGFR3	FGF19

FGFR3	FGF10

PTPRZ1	PTN

PTPRZ1	MDK

PTPRZ1	cntn1 ?

PTPRZ1	SEMA5

IL4R	IL4

IL4R	IL13

GUCY2C	GUCA2A

GUCY2C	GUCA2B

FAS	FASLG

CD40	CD40LG

CD40	LTB

ITGA3	RELN

ITGA3	DUSP18

ITGA3	LAMB1

ITGA3	LAMA2

ITGA3	LAMB3

ITGA3	LAMC2

ITGA3	LAMA3

ITGA3	LAMA5

ITGA3	LAMB2

ITGB7	MADCAM1

ITGB7	CDH1

ITGB7	VCAM1

ITGB7	FN1 

ITGB8	DUSP18

ITGB8	LAMB1

ITGB8	LAMA1

ITGB8	FN1

ITGB8	COL4A1

ITGB8	TGFB1

ITGB8	TGFB2

ITGB8	TGFB3

ITGB8	VTN

ACVR2A	INHBA

ACVR2A	INHBB

ACVR2A	BMP7

ACVR2A	NODAL

ACVR2A	INHA

ACVR2A	TDGF1

ACVR2A	GDF11

ACVR2A	BMP10

ACVR2A	GDF7

ACVR2A	BMP2

ACVR2A	BMP3

ACVR2A	BMP4

ACVR2A	BMP5

ACVR2A	BMP6

ACVR2A	BMP7

ACVR2A	BMP8

ACVR2A	BMP15

ACVR2A	INHBC

TNFRSF8	TNFSF8

EPHA2	EFNA1

EPHA2	EFNA3

EPHA2	EFNA5

EPHA2	EFNA4

EPHA2	EFNA2

EPHA3	EFNA5

EPHA3	EFNA1

EPHA3	EFNA2

EPHA3	EFNA3

EPHA3	EFNA4

EPHB2	EFNB1

EPHB2	EFNA5

EPHB2	EFNB3

EPHB2	EFNB2

AXL	GAS6

AXL	PROS1

CSF2RB	IL5

CSF2RB	IL3

CSF2RB	CSF2

RYK	WNT5A

RYK	WNT1

RYK	WNT3

RYK	WNT3a

TIE1	ANGPT1

TIE1	ANGPT4

TIE1	ANGPT2

FLT4	VEGFC

FLT4	FIGF

KDR	VEGF

KDR	SEMA6D

KDR	VEGFC

KDR	FIGF

KDR	VEGFA

FLT3	FLT3LG

BMPR1A	BMP2

BMPR1A	BMP4

BMPR1A	BMP6

BMPR1A	BMP7

BMPR1A	GDF6

BMPR1A	BMP10

BMPR1A	GDF7

BMPR1A	BMP3

BMPR1A	BMP5

BMPR1A	BMP8

BMPR1A	BMP15

BMPR1A	GDF5

ACVR1B	NODAL

ACVR1B	GDF11

ACVR1B	INHBA

ACVR1B	INHBB

ACVR1B	INHBC

ACVR1B	INHA

TGFBR1	GDF9

TGFBR1	TGFB1

TGFBR1	TGFB2

TGFBR1	TGFB3

ACVRL1	TGFB3

ACVRL1	BMP10

ACVRL1	GDF2

ACVRL1	TGFB1

TGFBR2	TGFB3

TGFBR2	TGFB1

TGFBR2	TGFB2

IFNGR2	IFNG

IL6ST	IL6

IL6ST	LIF

IL6ST	OSM

IL6ST	IL11

IL6ST	CNTF

IL6ST	CLCF1

IL6ST	CTF1

IL12RB1	IL12A

IL12RB1	IL12B

IL12RB1	IL23A

LIFR	LIF

LIFR	OSM

LIFR	NP

LIFR	CNTF

LIFR	CLCF1

LIFR	CTF1

TNFRSF4	TNFSF4

KIR2DL1	HLA-C

KIR2DL3	HLA-C

KIR3DL1	HLA-B

KIR3DL2	HLA-A

KIR2DS4	HLA-C

NOTCH1	JAG1

NOTCH1	JAG2

NOTCH1	NOV

NOTCH1	DLL1

NOTCH1	DLL4

NOTCH1	DLK1

NOTCH1	DLL3

CD151	DUSP18

CD151	LAMA5

CD151	LAMB1

CD151	LAMB2

IFNAR2	IFNA1

IFNAR2	IFNW1

IFNAR2	IFNB1

IFNAR2	IFNA4

IFNAR2	IFNA5

IFNAR2	IFNA16

IFNAR2	IFNA8

IFNAR2	IFNA14

IFNAR2	IFNA21

IFNAR2	IFNA13

IFNAR2	IFNA6

IFNAR2	IFNA7

IFNAR2	IFNA10

IFNAR2	IFNA17

PLXNA3	SEMA3F

PLXNA3	SEMA3A

ITGA8	FN1

ITGA8	NPNT

ITGA8	TNC

ITGA8	SPP1

ITGA8	TGFB1

ITGA8	TGFB3

ITGA8	VTN

EPHB3	EFNB1

EPHB3	EFNB2

EPHB3	EFNB3

EPHA5	EFNA5

EPHA5	EFNA1

EPHA5	EFNA2

EPHA5	EFNA3

EPHA5	EFNA4

EPHB1	EFNB2

EPHB1	EFNB1

EPHB1	EFNA3

EPHB1	EFNB3

EPHA4	EFNA5

EPHA4	EFNB3

EPHA4	EFNA1

EPHA4	EFNA3

EPHA4	EFNA2

EPHA4	EFNB2

EPHA4	EFNA4

EPHA4	EFNB1

PLXNA1	SEMA6D

PLXNA1	SEMA3A

PLXNA1	L1CAM

PLXNA1	SEMA3C

PLXNA1	SEMA3F

EDG2	LIPH

EDG2	ACP6

EDG2	ATX

IL13RA1	IL13

IL13RA1	IL4

VLDLR	RELN

VLDLR	APOE

VLDLR	TFPI

LRP2	AGT

NPSR1	NPS

IL9R	IL9

IL5RA	IL5

IL5RA	il3?

MC1R	ASIP

MC1R	POMC

ROR2	GDF5

ROR2	WNT5a

TNFRSF17	TNFSF13

TNFRSF17	TNFSF12

TNFRSF17	TNFSF13b

TEK	ANGPT1

TEK	ANGPT2

TEK	ANGPTL1

TEK	ANGPT4

NOTCH2	JAG1

NOTCH2	DLK1

NOTCH2	JAG2

NOTCH2	DLL1

NOTCH2	DLL3

NOTCH2	DLL4

ACVR1A	BMP6

ACVR1A	BMP7

ACVR1A	BMP3

ACVR1A	BMP4

ACVR1A	BMP5

ACVR1A	BMP8

ACVR1A	BMP10

ACVR1A	BMP15

ACVR1A	INHBC

ACVR1A	INHBA

ACVR1A	INHBB

ACVR1A	INHA

MST1R	MST1

TYRO3	GAS6

TYRO3	PROS1

TNFRSF9	TNFSF9

LRP1	A2M

LRP1	HSP90B1

LRP1	LRPAP1

LRP1	SERPINE2

LRP1	F2

LRP1	SERPINC1

LRP1	F2

LRP1	PLAT

LRP1	PLAU

LRP1	F9

LRP1	F8

LRP1	TFPI

LRP1	F7

LRP1	MMP9

LRP1	MMP13

LRP1	LTF

LRP1	PSAP

LRP1	C3

LRP1	SERPINE1

LRP1	SERPING1

LRP1	SERPINC1

LRP1	SERPINA1

IL10RB	IL28A

IL10RB	IL28B

IL10RB	IL29

IL10RB	IL10

IL10RB	IL22

IL10RB	IL26

IL10RB	IFNL1

DDR1	COL1A1

MERTK	GAS6

FZD5	WNT7A

FZD5	WNT5A

IL18R1	IL18

PTCH1	SHH

PTCH1	IHH

PTCH1	SHH

PTCH1	DHH

IL10RA	IL10

IL10RA	IL22

ITGA7	LAMA1

ITGA7	LAMA2

ITGA7	DUSP18

ITGA7	LAMB1

ACVR2B	NODAL

ACVR2B	GDF11

ACVR2B	LEFTY2

ACVR2B	BMP2

ACVR2B	BMP3

ACVR2B	BMP4

ACVR2B	BMP5

ACVR2B	BMP6

ACVR2B	BMP7

ACVR2B	BMP8

ACVR2B	BMP10

ACVR2B	BMP15

ACVR2B	INHBA

ACVR2B	INHBB

ACVR2B	INHBC

ACVR2B	INHA

ITGA9	PLG

ITGA9	ADAM12

ITGA9	ADAM15

ITGA9	VCAM1

BMPR2	BMP15

BMPR2	GDF6

BMPR2	GDF9

BMPR2	BMP2

BMPR2	GDF5

BMPR2	BMP10

BMPR2	GDF7

BMPR2	BMP4

BMPR2	BMP3

BMPR2	BMP5

BMPR2	BMP8

BMPR2	BMP7

FZD2	WNT4

FZD2	WNT5A

IL13RA2	IL13

KIR2DS1	HLA-C

ERBB4	NRG3

ERBB4	NRG2

ERBB4	BTC

ERBB4	NRG1

ERBB4	HBEGF

ERBB4	NRG4

ERBB4	EREG

ERBB4	HBEGF

EPHA7	EFNA1

EPHA7	EFNA2

EPHA7	EFNA3

EPHA7	EFNA5

EPHA7	EFNA4

EPHA7	EREG

NTRK2	BDNF

NTRK2	NTF5

NTRK2	NTF3

AMHR2	AMH

DDR2	COL1A1

DDR2	COL3A1

PLXNA2	SEMA3A

PLXNA2	SEMA6A

KREMEN2	DKK1

ACVR1C	NODAL

ACVR1C	ACTIVIN and BMP

IL31RA	IL31

ITGB2	CTGF

ITGB2	ICAM1

ITGB2	CYR61

ITGB2	ICAM5

ITGB2	ICAM4

ITGB2	ICAM3

ITGB2	ICAM2

ITGB2	VCAM1

ITGB2	C3

ITGB2	FGA

ITGB2	ICAM1

RXFP2	INSL3

RXFP2	RLN2

RXFP2	RLN1

TNFRSF25	TNFSF15

TNFRSF25	tnfsf12

IL22RA2	IL22

UNC5A	NTN4

UNC5A	NTN1

KREMEN1	DKK1

TNFRSF13C	TNFSF13B

NOTCH4	DLL4

NOTCH4	DLK1

NOTCH4	JAG1

NOTCH4	JAG2

NOTCH4	DLL1

NOTCH4	DLL3

OSMR	OSM

OSMR	IL31

IL12RB2	IL12A

IL12RB2	IL12B

KIR2DL4	HLA-G

KIR2DL4	HLA-C

SMO	SHH

RTN4R	RTN4

RTN4R	OMG

FZD8	WNT1

FZD8	WNT3

EDA2R	EDA

IL22RA1	IL22

IL22RA1	IL20

IL22RA1	IL24

NMUR1	NMS

NMUR1	NMU

EDG4	LIPH

RXFP1	RLN2

RXFP1	RLN3

RXFP1	RLN1

PLXNB3	SEMA5A

TNFRSF12A	TNFSF12

FZD3	WNT1

TNFRSF10D	TNFSF10

CRLF1	CNTF

CRLF1	CLCF1

FZD4	WNT5A

FZD4	NDP

FZD10	WNT7B

NOTCH3	JAG1

NOTCH3	JAG2

NOTCH3	DLK1

NOTCH3	DLL1

NOTCH3	DLL3

NOTCH3	DLL4

EDAR	EDA

FZD1	WNT7B

PTCH2	DHH

PTCH2	SHH

ROBO1	SLIT1

ROBO1	SLIT2

ROBO1	NCAM1

ROBO1	SLIT3

TNFRSF11A	TNFSF11

IL1R2	IL1A

IL1R2	IL1B

csf3r	csf3

PTPRB	MDK

PTPRB	PTN

CCXCR1	SCYC1

CCXCR1	SCYC2

CCPB2	SCYA11

CCPB2	SCYA4

CCPB2	ccl5

CCPB2	SCYA2

CCPB2	SCYA8

CCPB2	CCL7

CCPB2	CCL13

CCR4	CCL5

CCR4	SCYA17

CCR4	SCYA22

HTR3C	TPH …

HTR3B	TPH …

HTR3D	TPH …

HTR3E	TPH …

CHRNA1	CHAT

CHRNA2	CHAT

CHRNA3	CHAT

CHRNA4	CHAT

CHRNA5	CHAT

CHRNA6	CHAT

CHRNA7	CHAT

CHRNA9	CHAT

CHRNA10	CHAT

CHRNB2	CHAT

GABRA1	HDC

ADORA1	adenosine

ADORA2A	adenosine

ADORA2B	adenosine

ADORA3	adenosine

APLNR	APLN

GPBAR1	bile acid

CNR1	canabis receptor

CNR2	canabis receptors

CXCR2	il8

CXCR2	CXCL3

CXCR2	MGSA

CXCR2	NAP-2

CXCR4	CXCL12

CXCR4	UBC

ACKR2	CCL2

ACKR2	CCL3

ACKR2	CCL3L1

ACKR2	CCL4

ACKR2	CCL5

ACKR2	CCL7

ACKR2	CCL8

ACKR2	CCL11

ACKR2	CCL13

ACKR2	CCL17

ACKR2	CCL22

ACKR2	CCL23

ACKR2	CCL24

ACKR2	SCYA2

ACKR2	SCY3

ACKR2	SCYA5

ACKR2	SCYA7

ACKR3	CXCL11

ACKR3	CXCL12

CCRL2	CCL19

CCRL2	RARRES2

C5AR2	C3a

C5AR2	C4a

C5AR2	C5a

FPR2	formylethionyl

FPR3	formylethionyl

LHCGR	LH

LHCGR	HCG

GPR55	LPI

GPER1	cyp19a1

HCAR1	orphan

HCAR2	Bind externe elements

HCAR3	Bind externe elements

KISS1R	kiss1

LTB4R	LTC4

LTB4R	LTD4

LTB4R	LTE4

LTB4R	2-HETE

LTB4R2	LTC4

LTB4R2	LTD4

LTB4R2	LTE4

LTB4R2	2-HETE

CYSLTR1	LTC4

CYSLTR1	LTD4

CYSLTR1	LTE4

CYSLTR1	2-HETE

CYSLTR2	LTC4

CYSLTR2	LTD4

CYSLTR2	LTE4

CYSLTR2	2-HETE

OXER1	LTC4

OXER1	LTD4

OXER1	LTE4

OXER1	2-HETE

S1PR1	PSPH

S1PR2( EDG5)	PSPH

S1PR3 (EDG3)	PSPH

S1PR4(EDG6)	PSPH

S1PR5(EDG8)	PSPH

MCHR1	PMCH

NPFFR1	NPFF

NPFFR1	NPAF

NPY6R	PPY

NPY6R	NPY

NPY6R	PYY

NPY4R	NPY

NPY4R	PYY

OPRD1	PDYN

OXGR1	OGDH

P2RY1	ATP

P2RY2	ATP

P2RY4	ATP

P2RY6	ATP

P2RY11	ATP

P2RY11	ADP

P2RY12	ATP

P2RY13	ATP

P2RY14	ATP

PTGDR2	PTGD2

PTGER1	PTGES3

PTGER1	PTGS2

PTGER1	PTGIS

PTGER1	PTGD2

PTGER2	PTGES3

PTGER2	PTGS2

PTGER2	PTGIS

PTGER2	PTGD2

PTGER3	PTGES3

PTGER3	PTGS2

PTGER3	PTGIS

PTGER3	PTGD2

PTGER4	PTGES3

PTGER4	PTGS2

PTGER4	PTGIS

PTGER4	PTGD2

PTGIR	PTGIS

QRFPR	QRFP

SUCNR1	SDHA

THRH	amines, amphetamines, dopamine, serotonine, histamine

TAAR1	amines, amphetamines, dopamine, serotonine, histamine

CASR	amines, amphetamines, dopamine, serotonine, histamine

GPRC6A	amines, amphetamines, dopamine, serotonine, histamine

GRM1	GLUTAMATES receptors

GRM2	GLUTAMATESreceptors

GRM3	GLUTAMATESreceptors

GRM5	GLUTAMATESreceptors

GRM6	GLUTAMATESreceptors

GRM7	GLUTAMATES receptors

GRM8	GLUTAMATES receptors

INSRR	INS

INSRR	PTEN

EPHA10	EFNA1

EPHA10	EFNA2

EPHA10	EFNA3

EPHA10	EFNA4

EPHA10	EFNA5

LTK	ORPHAN

ROR1	ORPHAN

PTK7	WNT

IL23R	IL23

TSLPR	TSLP

IL20RA	IL19

IL28RA	IFNL2

IL28RA	IFNL3

IL18RAP	IL18

IL1RL1	IL33

IL1RL2	IL18

IL17RC	IL17

IL17RD	IL17

IL17RE	IL17

THRA	TSH

THRA	TPO

THRB	TSH

THRB	TPO

cd28	cd80

cd28	cd86

cd28	ICOSLG

CTLA4	CD80

CTLA4	CD86

CD2	LFA-3

CD2	CD48

AGC1	SEMA5

VCAN	HAS2

NCAN	HAS2

BCAN	HAS2

CSPG4	colalpha

KIR2DL2	HLA-C

kir2dl5a	HLA-C

KIR2DL5B	HLA-C

KIR2DS2	HLA-C

KIR2DS3	HLA-C

KIR2DS5	HLA-C

KIR3DL3	HLA-C

KIR3DS1	HLA-C

LILRA1	HLA

LILRA2	HLA

LILRA3	HLA

LILRA4	HLA

LILRA5	HLA

LILRA6	HLA

LILRB2	HLA

LILRB3	HLA

LILRB4	HLA

LILRB5	HLA

EPHA8	EFNB3

TNFRSF1A	TNFSF2

TNFRSF1A	TNFSF1

TNFRSF1B	TNFSF2

TNFRSF1B	TNFSF1

FLT4	VEGFD

KDR	VEGFD

EPHA10	EFNB1 

EPHA10	EFNB2 

EPHA10	EFNB3 

GLRA1	SHMT 

GLRA2	SHMT 

GLRA3	SHMT 

GLRA4	SHMT 

GLRB	SHMT 

GABRA2	GAD 

GABRA1	GAD 

GABRA4	GAD 

GABRA5	GAD 

GABRA6	GAD 

GABRA1	GAD
